# Anthocyanins: From Natural Colorants to Potent Anticancer Agents

**DOI:** 10.1002/fsn3.70232

**Published:** 2025-05-02

**Authors:** Muhammad Maaz, Muhammad Tauseef Sultan, Ahmad Mujtaba Noman, Shehnshah Zafar, Naima Tariq, Muzzamal Hussain, Muhammad Imran, Ahmed Mujtaba, Tadesse Fenta Yehuala, Ehab M. Mostafa, Samy Selim, Soad K. Al Jaouni, Suliman A. Alsagaby, Waleed Al Abdulmonem

**Affiliations:** ^1^ Department of Human Nutrition, Faculty of Food Science and Nutrition Bahauddin Zakariya University Multan Pakistan; ^2^ TIMES Institute Multan Multan Pakistan; ^3^ Department of Food Science and Technology, Faculty of Food Science and Nutrition Bahauddin Zakariya University Multan Pakistan; ^4^ Department of Food Science Government College University Faisalabad Faisalabad Pakistan; ^5^ Department of Food Science and Technology University of Narowal Narowal Pakistan; ^6^ Department of Food Sciences and Technology, Faculty of Engineering Sciences and Technology Hamdard University Islamabad Campus Islamabad Pakistan; ^7^ Faculty of Chemical and Food Engineering Bahir Dar Institute of Technology, Bahir Dar University Bahir Dar Ethiopia; ^8^ Department of Pharmacognosy, College of Pharmacy Jouf University Sakaka Saudi Arabia; ^9^ Department of Clinical Laboratory Sciences, College of Applied Medical Sciences Jouf University Sakaka Saudi Arabia; ^10^ Department of Hematology/Oncology, Yousef Abdulatif Jameel Scientific Chair of Prophetic Medicine Application, Faculty of Medicine King Abdulaziz University Jeddah Saudi Arabia; ^11^ Department of Medical Laboratory Sciences, College of Applied Medical Sciences Majmaah University Al‐Majmaah Saudi Arabia; ^12^ Department of Pathology, College of Medicine Qassim University Buraidah Saudi Arabia

**Keywords:** anthocyanins, bioavailability, mitochondrial dysfunction, nano‐delivery, oncogenes, pro‐inflammatory cytokines

## Abstract

Cancer is a prevalent global disease affecting ~20 million individuals, and this burden causes the death of ~9.7 million people in 2024. The prevalence rate is continuously increasing due to exposure to harmful environmental and occupational contaminants (toxins and chemicals), compromised immune response, genetic modifications, and poor lifestyle and dietary practices. The management of cancer is challenging and demands cost‐effective and safe therapeutic strategies. This review accentuates the anticancer potential of anthocyanins and its associated underlying mechanism. Anthocyanins, the active components extracted from grapes, berries, black chokeberries, eggplants, black currants, sweet cherries, strawberries, black grapes, plums, and red onions, hold antioxidant and anti‐inflammatory potential. The bioavailability of anthocyanins is a crucial factor in imposing their anticancer effect, and this bioavailability can be improved by microbial phenolic catabolites, provision of α‐casein, and nano delivery systems. Anthocyanins hinder cell migration, invasion, and proliferation by inducing apoptosis, suppressing cell cycle at G0/G1, S, or G2/M stages, and modulating signaling pathways such as apoptotic cascades, PI3K/Akt, MAPK, and NF‐κB. Moreover, anthocyanins downregulate oncogenes (*Bcl‐2*, *MYC*, and *HER2*) and improve the activity of tumor suppressor genes (*TP53*, *BRCA1*, and *RB1*). Anthocyanins, particularly cyanidin‐3‐*O*‐glucoside, suppress inflammation and production of pro‐inflammatory cytokines (COX‐2, TNF‐α, and IL‐6) in colorectal cancer and hepatocellular carcinoma. Moreover, it causes cell cycle inhibition and mitochondrial dysfunction in ovarian and cervical malignancies. Although pre‐clinical studies have proved anticancer activities, further clinical trials are required to validate its therapeutic impact and standard dose regimens.

## Graphical abstract

Anthocyanin bioavailability has a significant contribution to imposing therapeutic effects on abnormal cells. Anthocyanins hinder cell migration, invasion, and proliferation by inducing apoptosis, suppressing the cell cycle at G0/G1, S, or G2/M stages, and modulating oxidative stress and signaling pathways such as apoptotic cascades, PI3K/Akt, MAPK, and NF‐κB. Moreover, anthocyanins downregulate oncogenes (Bcl‐2, MYC, HER2) and improve the activity of tumor suppressor genes (TP53, BRCA1, and RB1). The antioxidant activity of anthocyanins further inhibits the invasion of cancerous cells. Mainly, cyanidin‐3‐O‐glucoside suppresses inflammation and the production of pro‐inflammatory cytokines (COX‐2, TNF‐α, and IL‐6) in colorectal and hepatocellular carcinoma and causes cell cycle inhibition and mitochondrial dysfunction in ovarian and cervical malignancies. This review accentuates the anticancer potential of anthocyanins and its associated underlying supporting mechanism.

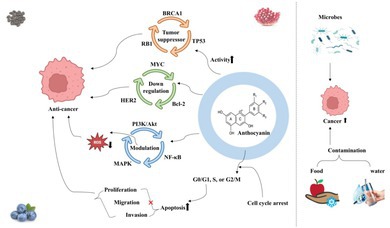

## 1 Introduction

Humans, plants, and animals have had mutual interaction for centuries for the maintenance of the environment and individual health status (Bosch et al. 2024). The plants and animals have been consumed in healthcare systems to prevent the prevailing disorders. In addition, humans also consume plants and animals to satisfy their appetite and to fulfill their dietary requirements (Oda et al. 2024). The utilization of plants and plant‐derived products has been significantly reduced with the progression of industrial frameworks, which ultimately disturbs the dietary patterns of individuals (Dehelean and Pînzaru 2024). A laborious and hectic routine triggers individuals to consume processed and ready‐to‐eat fast foods containing abundant fats and sugars. These foods produce more free radicals such as reactive oxygen species (ROS), reactive nitrogen species (RNS), nitric oxide (NO), and hydrogen peroxide (H_2_O_2_), which lead to a deteriorated cell growth cycle and enhanced necrosis process (Samanci et al. 2024). The prolonged abundant production of free radicals and limited antioxidant consumption develop malignant tumors or cancerous cells that proliferate to other cells, damaging their performance (Munteanu and Schwartz 2024). Cancer is a complicated and malignant form of disorder that compromises the health status of ~20 million individuals and enhances the mortality rate (worldwide, ~9.7 million deaths are recorded due to cancer). Poor dietary choices, environmental conditions (toxins, chemicals, and seasonal variations), sedentary lifestyles, genetic variations, and occupational factors contribute to severe cancer pathogenesis via increasing oxidative damage (Bhol et al. 2024). Although diverse chemotherapeutic drugs are available in the market, the adverse effects, for example, anemia, appetite suppression, loss of bone mineral density, fatigue, nausea, and vomiting, of these drugs have urged scientists to develop alternative and novel therapeutic agents that are safe to use as well as effective against oxidative stress‐induced disorders. Antioxidant plants and their bioactive metabolites, such as anthocyanins, effectively slow the progression of various disorders, such as diabetes, cancer, gastrointestinal, inflammatory, and neurodegenerative disorders (Muscolo et al. 2024). Anthocyanin is a red, blue, orange, and purple‐colored bioactive compound that is extracted from mulberries, black chokeberries, eggplants, black currants, sweet cherries, strawberries, black grapes, plums, and red onions (Yücetepe et al. 2024). It can be employed in nutraceutical and functional food industries to acquire remedial supplements because of its adaptogenic properties. This review has comprehensively provided the anticancer potential of anthocyanins against different types of cancer, including breast, prostate, lung, liver, ovarian, and cervical cancers, by highlighting possible signaling pathways (MAPK, NF‐kB, and PI3k/Akt) and gene expression (*TP53, BRCA1*, and *BCL‐2*). Moreover, this current review focuses on the stability and bioavailability of anthocyanins. The novel strategies to enhance anthocyanins' stability are the limelight of this review.

The data for this review were collected from Google Scholar, PubMed, and Science Direct covering recent data (2015–2025). Multiple keywords, such as bioavailability, anthocyanin's bioavailability, cancer and anthocyanins, breast cancer and anthocyanins, uterine cancer and anthocyanins, cervical cancer and anthocyanins, oral cancer and anthocyanins, renal cancer and anthocyanins, were used to search the data.

## 2 Bioavailability of Anthocyanins

The bioavailability of anthocyanins is the critical factor, which can directly or indirectly impact its therapeutic potential. Different factors, such as environmental conditions, climatic variations, consumption, intestinal bioactivity, and microbial populations, can influence the bioavailability of anthocyanins (Gonçalves et al. 2021; Han et al. 2019). Various studies were conducted to improve the bioavailability of anthocyanins from different plant sources, and factors associated with their bioavailability were also studied. Besides these factors, the delivery route is another significant aspect which can affect bioavailability. Regarding this, the bioavailability of anthocyanins isolated from bilberry extracts was investigated in rats by providing oral and intravenous (IV) bilberry extract (400 mg/kg), and it was found that the IV route enhanced plasma anthocyanins up to the level of 1.2 μM within 15 min (Ichiyanagi et al. 2006). Similarly, Charron et al. (2009) evaluated the bioavailability of acylated and non‐acylated anthocyanins by providing 50, 100, and 250 mL of purple carrot juice after 8 h of intake. It has been revealed that despite having maximum anthocyanin contents (76%) in acylated anthocyanins, the bioavailability is relatively reduced compared to the non‐acylated anthocyanins (having 4 times more bioavailability). Moreover, the intestinal bioavailability of anthocyanins from bilberries was investigated in humans with and without colon (artificial gut). It has been observed that the absorbed anthocyanins were in the form of glucuronides where the intestine contributed a major role and recorded the absorbed concentration of 27 nmol/L. However, a comparatively lower quantity of anthocyanins (13 nmol/L) was absorbed into the circulation in the absence of colon, demonstrating the role of colon in the absorption of anthocyanins (Mueller et al. 2017). Previously, Xie et al. (2016) assessed anthocyanins' bioavailability and polyphenolic metabolites in six individuals by providing them with Aronia berry extract (~500 mg). It has been shown that microbial phenolic catabolites have increased tenfold anthocyanins and its metabolites bioavailability, such as protocatechuic acid (0.005 ± 0.001 μg/mL and 0.431 ± 0.075 mg/mg) and cyanidin‐3‐O‐glucoside (0.059 ± 0.024 μg/mL and 0.010 ± 0.006 mg/mg) in plasma and urine respectively after 24 h. Kirakosyan et al. (2015) conducted a study in rats to determine the bioavailability of anthocyanins in targeted bladder, kidney, liver, heart, brain, and retroperitoneal fat. It was depicted that cyanidin 3‐glucosylrutinoside was found in the highest concentration (2339 ρg/g of tissues) within the bladder and imposed beneficial effects. In another study, the bioavailability of borage extracted anthocyanins was determined, and it was found that the bioavailability of cyanidin‐3‐glucoside, cyanidin‐3‐rutinoside, pelargonidin‐3‐glucoside, and cyanin chloride was 71%, 77%, 80%, and 90%, respectively, with the highest gastrointestinal stability (99%) shown by cyanidin‐3‐glucoside (Zannou et al. 2021).

The bioavailability of anthocyanins is improved and decreased by other bioactive compounds depending on the chemical composition and intrinsic nature of the compounds. Therefore, Lang et al. (2021) conducted a study investigating the impact of α‐casein on anthocyanins bioavailability and found that the bioavailability has significantly increased (~1.5 to 10 folds) by α‐casein administration. Moreover, da Silva et al. (2021) reported that natural deep eutectic solvent‐extracted anthocyanins from blueberry have more bioavailability (~140%) than organic solvent‐extracted anthocyanins. The delivery route contributes to the accessibility of anthocyanins to perform their metabolic activity within the body. Nanotechnology is an emerging field that develops nanodrugs and nano‐emulsions to improve drugs or supplements' bioavailability. Therefore, the bioactivities, stability, and bioavailability were assessed by providing anthocyanins through formulating nano‐capsules. The results revealed that anthocyanins' bioavailability was improved, revealing oral, gastric, and intestinal stability (dos Santos et al. 2022; Salah et al. 2020). Blanching, high‐pressure processing, co‐pigmentation with coloring compounds, flavonoids, metal ions, whey protein, carbohydrates, and nanoliposomes are the different methods that contribute to enhance the stability of anthocyanins (Al‐Khayri et al. 2022; Mueller et al. 2018). Moreover, Amararathna et al. (2022) utilized anthocyanins‐enriched haskap berries to encapsulate in polyethylene glycol‐poly, maltodextrin, and carboxymethyl chitosan. It has been revealed that anthocyanins efficiency is relatively higher (10%) in carboxymethyl chitosan as compared to polyethylene glycol‐poly and maltodextrin. Furthermore, the release of bioactive compounds, target drug delivery, drug availability, and stability are controlled by the encapsulated delivery systems (Yuan et al. 2023). Anthocyanins are protected from the gastric environment by synthesizing calcium alginate and chitosan beads (Kanokpanont et al. 2018). Chitosan and cellulose microcapsules improve the stability of anthocyanins by attenuating the influence of oxygen, light, and temperature (Wang et al. 2017).

## 3 Antioxidant Potential

Oxidative stress disturbs routine activities and lifestyle by imposing various disorders, including mental fatigue, memory loss, insomnia, wrinkles, and skin deterioration, leading to further chronic anomalies (Alzheimer, Parkinson, early aging, diabetes mellitus, obesity, atherosclerosis, and hypertension) among individuals (Diniz et al. 2024). Free radicals are the reactive species that are majorly involved in developing cardiovascular, respiratory, and neurodegenerative disorders (Mustafa 2024; Jebir and Mustafa 2022). Various studies have been conducted over the past few years to validate the antioxidant profile of plants and their active metabolites, such as flavonoids, coumarins, and terpenoids (Hu et al. 2023). These metabolites aid in maintaining a balance between oxidative stress and antioxidants to ameliorate the severity of diseases (Mustafa et al. 2025; Vázquez‐Lorente et al. 2024). The antioxidant property of the compounds or metabolites depends on compound composition, structure, environment (temperature, pH, and humidity), and processing techniques (Younes and Mustafa 2024; Germán‐Soto et al. 2024). The antioxidant profile of anthocyanins has been studied and described here. DPPH and ABTS assays revealed that the antioxidant activity of anthocyanins has been significantly attenuated by various treatments, including illumination, sucrose, heat, and vitamin C (Zang et al. 2021). However, ultraviolet irradiation improved the neutralizing activity of sweet cherries isolated anthocyanins (Zhang, Jiang, et al. 2021; Zhang, Yang, et al. 2021). DPPH and lipid peroxidation inhibition assays were conducted to investigate the antioxidant potential of anthocyanins (A1, A2, A3, and A4). It was observed that A3 has relatively higher free radical scavenging potential (~90%) than A1 (84%), A2 (79%), A4 (82%), and resveratrol (74%). Moreover, the lipid peroxidation inhibition assay showed a higher scavenging potential of resveratrol than the anthocyanins. A2 and A3 had 87% LDL inhibition potential, while A4 and A1 had 74% and 71%, respectively (Ma et al. 2020). Later, Zannou and Koca (2022) determined the antioxidant potential of anthocyanins isolated from blackberries using natural deep eutectic solvents (NaDEs). It has been revealed that NaDEs have comparatively higher antioxidant potency than other solvents (ethanol, methanol, and distilled water), with CHCI (choline chloride and citric acid) showing higher DPPH (68.7 mmol TE/g), TARXYL (tartaric acid and xylitol) having higher FRAP (83.08 mmol ISE/g), and ACSOR (acetic acid and sorbitol), and having high FCA/iron chelating activity (4687 mg TE/100 g) values. Anthocyanins extracted from raspberry using supercritical water extraction (SWE) depicted elevated total phenolic contents (5.9 mg/g), while hot water extracted and methanol extracted anthocyanins have TPC of 3.1 and 3.8 mg/g, respectively. Furthermore, the antioxidant potential assessed by DPPH and ABTS analysis revealed a higher scavenging potential of SWE (83% and 66%) as compared to hot water and methanol extraction (Wang et al. 2021).

### 3.1 In Vitro Studies

Various pre‐clinical trials have evidenced the cancer‐alleviating mechanism of anthocyanins in human cell lines. The anticancer potential of anthocyanins was evaluated against human colorectal cancer cells (SW480 and SW620) by providing 10%–40% (v/v) of Andean berry's aqueous extract for 1–3 days. It has been observed that the cell growth phase was inhibited at the S and G2/M phase and G0/G1 phase for SW480 and SW620, respectively, which results in reducing affected cell proliferation without causing mitochondrial disturbance (Arango‐Varela et al. 2021). Olejnik et al. (2018) isolated anthocyanins from *Ribes nigrum* L. fruit extract and investigated its anti‐proliferative effect against human colon cancer (HT‐29 & NCM460) cells. A dose‐dependent relationship has been observed, which showed 10% cell viability (EC10) at 3.03 and 5.36 mg/mL for respective HT‐29 and NCM460 cell lines. In comparison, 50% inhibition (EC50) was recorded at 6.19 and 14.17 mg/mL, respectively. Moreover, the EC90 for HT‐29 and NCM460 was 12.65 and 37.59 mg/mL, respectively. Hibiscus anthocyanins extracts have also been shown to possess a protective effect against human colorectal cancer cells in a dose‐and time‐dependent response by modulating AMP‐activated kinase in inducing apoptosis (Tsai et al. 2023). Additionally, Chen et al. (2023) investigated the impact of protein acetylation of black raspberry anthocyanins in reducing colorectal cancer progression. It has been revealed that black raspberry improved the acetylation process by reducing Sirtuin 1 and increasing MOF and EP300 expression. Moreover, its treatment reduced colorectal cancer proliferation by modulating the NF‐κB pathway *and Bax* expression while suppressing *Bcl‐2*, *cyclin‐D1*, *c‐myc*, and *NLRP3* expression.

However, Kim, Paramanantham, et al. (2020) and Kim, Jegal, et al. (2020) employed anthocyanins extracted from *Vitis coignetiae Pulliat* in a trial to determine the anti‐proliferative potential in attenuating human hepatocellular carcinoma (Hep3b). It has been evident that *Vitis coignetiae Pulliat* significantly suppressed cell proliferation, migration, and invasion at 100 μg/mL concentrations via inactivating the NF‐κB pathway. Purple sweet potatoes constituting acylated anthocyanins possess anti‐cancerous and antimutagenic properties. Guo et al. (2022) conducted a study to demonstrate the underlying mechanism of purple sweet potato against acute lymphoblastic leukemia. It was revealed that purple sweet potato significantly ameliorated cell proliferation and the apoptotic pathway by improving intracellular calcium levels in T‐ALL cells and targeting the NFAT5 and p38/NF‐κB/Bcl‐2/caspase‐3 axis. Afterward, Fan et al. (2022) extracted anthocyanins from *Lycium ruthenicum Murray* and evaluated its impact on the metastasis of liver carcinoma (HepG2). It has been evident that cell viability, migration, and metastasis are attenuated by *Lycium ruthenicum Murray*, which stimulated the apoptotic and cell cycle inhibitory processes. Moreover, modulation of *p62*, *LC3‐II/LC3‐I*, *beclin‐1*, and p‐AMPK expressions, as well as suppression of p‐mTOR expression, was revealed by anthocyanins.

## 4 Anticancer Perspectives

Anthocyanins have anticancer properties against the most prevalent cancers, that is, breast, brain, oral, esophageal, gastric, pancreatic, hepatic, colorectal, bladder, cervical/uterine, and prostate cancers.

## 5 Breast Cancer

Breast cancer is the most frequent and prevalent cancer among women throughout the world, which accounts for approximately 15% of all cancer‐associated mortalities. Medicinal plants and their metabolites are being studied to evaluate their anticancer properties. For instance, the antiproliferative potency of *Eucalyptus camaldulensis* was confirmed by Huang et al. (2022) against MCF‐7 breast cancer cells. Layosa et al. (2021) investigated the antimutagenic potential of dark sweet cherries enriched with anthocyanins against breast cancer (MDA‐MB‐453) cell lines, and it was found that anthocyanins ameliorated Akt and PLCγ‐1 modulation to suppress cell migration and invasion. Additionally, anthocyanins stimulated caspase‐8 cleavage, MAPK, ERK1/2, and p38 activation to promote the apoptotic pathway, thereby showing a protective effect against MDA‐MB‐453 cells. Previously, Lage et al. (2020) demonstrated that phenolic acids, flavonols, and proanthocyanidins showed cell viability and an antiproliferative effect by downregulating metastatic markers, including Sp1, Sp4, and VCAM‐1. Breast cancer cells, especially MCF‐7, are more sensitive to cisplatin than other breast cancerous cells. Therefore, anthocyanins from *Vitis coignetiae Pulliat* have been investigated to confirm the breast cancer inhibitory potential in MCF‐7 cell lines. It has been evident that anthocyanins inactivated AKT, anti‐apoptotic proteins, and the XIAP pathway while modulating p‐NF‐κB, p‐lκB, and PARP‐1 cleavage in MCF‐7 cells (Paramanantham et al. 2020). Similarly, Tan et al. (2020) extracted anthocyanins from grape skins and proved an antimutagenic effect against MCF‐7 tumor cells.

Moreover, Aqil et al. (2021) revealed that oral provision of anthocyanins enriched bilberry or blueberry suppressed the growth of MDA‐MB‐231 cell lines by downregulating the NF‐κB pathway. The cell viability assay was conducted to investigate the cytotoxic effect of isolated anthocyanins in black carrots encapsulated in halloysite nanotubes against MCF‐7 breast cancer cells. The results showed that tumor growth was suppressed two times with halloysite nanotubes containing anthocyanins (Hamedi and Koosha 2020). Anthocyanins are isolated from fruits and vegetables such as red cabbage, black beans, eggplant, red onion, raspberry, blueberry, blackberry, pomegranate, and red grapes. Catacutan et al. (2024) evaluated the antitumorigenic potential of anthocyanins from these fruits and vegetables on the propagation of breast cancer cells, and it has been concluded that cell invasion, cell migration, and proliferation of MCF‐7 and MDA‐MB‐231 cells are significantly attenuated by blueberry anthocyanins.

**FIGURE 1:**
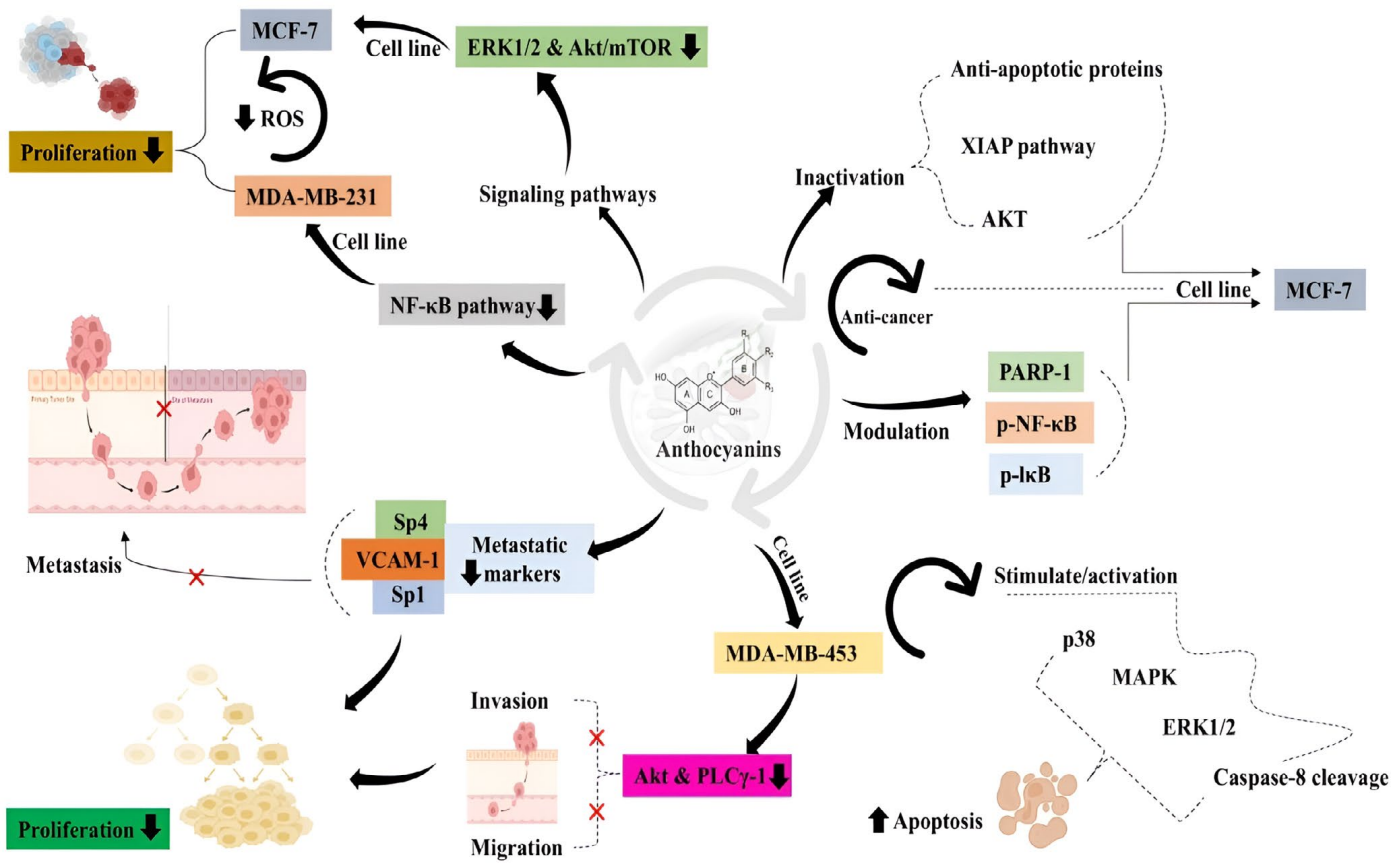
Breast cancer protective mechanism of anthocyanins.

Bracone et al. (2021) conducted a randomized, controlled, double‐blind clinical study to assess the protective mechanism of anthocyanins against radiotherapy‐induced skin toxicity by supplementing 125 mg of anthocyanins thrice a day for 21 to 35 days among 193 individuals. A moderate variation in skin appearance was observed in the anthocyanins group compared to placebo groups. Silveira Rabelo et al. (2022) depicted that anthocyanin has shown a protective mechanism for breast cancer by promoting the apoptotic process, caspase‐3 cleavage, and PARP suppression. The results also revealed that anthocyanins suppressed the ERK1/2 and Akt/mTOR signaling pathways. Later, Eze et al. (2024) and Wang et al. (2023) uncovered the antiproliferative potential of anthocyanins against MCF‐7 and MDA‐MB‐231 cancer cell lines by modulating the apoptotic pathway as well as by suppressing reactive oxygen species and oxidative stress. Awad et al. (2024) prepared anthocyanins‐loaded carbon nanotubes coated with chitosan and folic acid and explored their inhibitory potential for MCF‐7 cells. The outcomes showed a protective effect by reducing tumor migration, invasion, and inflammation (Figure 1).

## 6 Liver Cancer

Liver cancer is the sixth most frequent type of cancer worldwide and is ranked third on the scale of mortality. Various factors, including environmental modifications, food and water contamination, and microbial populations, are involved in the pathogenesis of hepatocellular carcinoma (McGlynn et al. 2024; Chen et al. 2024). Anthocyanin, a bioactive compound, possesses anticancer potential. Therefore, multiple studies have been conducted to validate its impact on liver carcinoma. For instance, Liu et al. (2023) synthesized xanthone and anthocyanins nano‐emulsions to investigate their potential in attenuating liver cancer (HepG2) cell lines. It has been observed that xanthone nano‐emulsions had relatively more cancer‐suppressing effects than their extract, with IC50 values of 5.78 and 6.23 μg/mL, respectively. In contrast, nano‐emulsions of anthocyanins have no significant impact on HepG2 cells. Additionally, the in vivo trial has been conducted to evaluate the protective mechanism of black raspberry anthocyanins in acute and subacute alcoholic liver disease. A significant reduction in AST, ALT, and LDL levels was observed in both acute and subacute alcoholic liver disease mice by the administration of high‐dose black raspberry anthocyanins. Moreover, anthocyanin's cytotoxic potential against HepG2, Hep3B, and t‐HSC/Cl‐6 was revealed by apoptosis induction, *Bax* activation, *Bcl‐2* suppression, and the modulation of cleaved caspase‐3, cleaved caspase‐9, and cleaved PARP (Xiao et al. 2021).

Kim, Paramanantham, et al. (2020) and Kim, Jegal, et al. (2020) explored the antimetastatic activity of extracted anthocyanins from *Vitis coignetiae Pulliat* against Hep3B cells. The results showed that ~100 μg/mL of anthocyanins downregulated cell invasion and proliferation. The suppressive mechanism of anthocyanins in the xenograft model of Hep3B human liver carcinoma cells was observed due to the inactivation of NF‐κB and Ki67 activity. *Lycium ruthenicum Murray* is an efficient source of anthocyanins employed by Fan et al. (2022) to assess the anti‐cancerous activity in hepatocellular carcinoma. The antitumor potential of anthocyanins was revealed by reducing cell viability, migration, and invasion. Moreover, improved expression of *Beclin‐1*, *p62*, *p‐AMPK*, and *LC3‐II/LC3‐I* demonstrated the liver cancer protective effect. Awad et al. (2024) depicted the cytotoxic potential of encapsulated anthocyanins loaded with carbon nanotubes coated with chitosan and folic acid by elevating *Bax* expression and alleviating *Bcl‐2* expression. The anti‐proliferative effect of anthocyanins was also reported against hepatocellular carcinoma cells by Romualdo et al. (2021). They demonstrated that anthocyanins lowered Ki‐67, TNF‐α, and lipid peroxidation levels while modulating glutathione and catalase activity. Later, Du et al. (2023) also validated the hepatic cancer protective effect of anthocyanins, which was depicted by the 100 mg/kg supplementation and 200 mg/kg.

**FIGURE 2:**
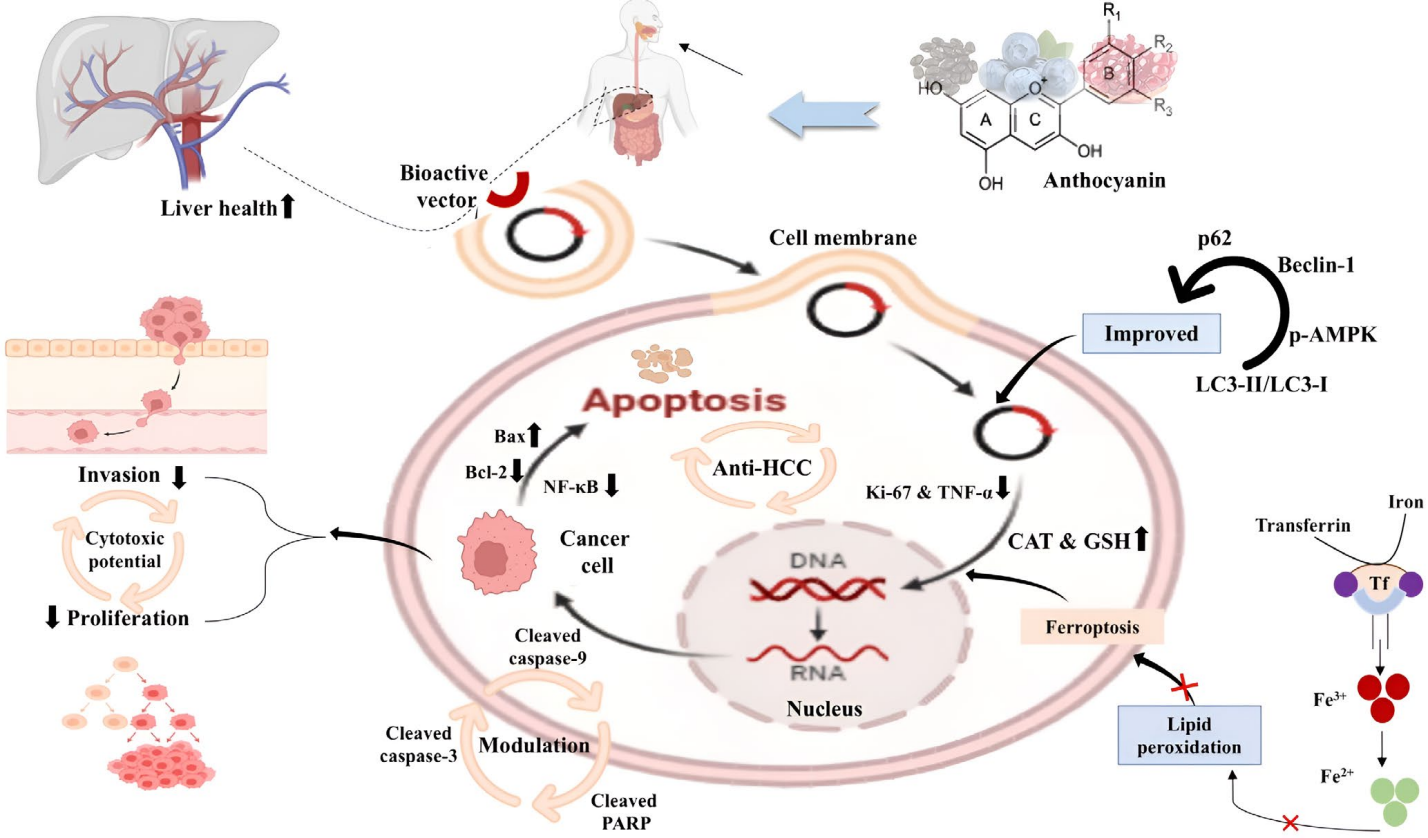
Underlying mechanism of anthocyanins against hepatic carcinoma.

Zhao et al. (2023) isolated anthocyanins from Gardenblue through an ultrasound‐assisted solvent extraction method and determined their potential against HepG2 cell lines, revealing inhibitory potential with an IC50 value of 23.57 μg/mL. Additionally, the antimetastatic activity of anthocyanins was improved when combined with cisplatin and doxorubicin, presenting the IC50 value of 0.02 μg/mL. The induction of cell apoptosis and disruption of DNA were observed, which arrested the cell growth phase and propagation. Cyanidin‐3‐glucoside, a polar anthocyanin, was used to assess its impact on the cell cycle in diethylnitrosamine/2‐acetylaminofluorene induced hepatic carcinomic Wistar rats by administering 10, 15, and 20 mg/kg/day for 4 days a week for 4 months. The results have suppressed hepatic carcinoma by attenuating alpha‐fetoprotein, non‐coding RNA MALAT1, and tubulin gamma 1 mRNA and by modulating miR‐125b, which is involved in the cell cycle (Matboli et al. 2021). Zhang, Jiang, et al. (2021) demonstrated that the supplementation of lingonberry anthocyanins (25–100 μg/mL) inactivated hepatic stellate cells and CCL4‐induced liver fibrosis by suppressing hydroxyproline, malondialdehyde (MDA), and TGFβ/Smad/ERK signaling pathway, as well as by improving superoxide dismutase (SOD), glutathione peroxidase (GSH‐Px), and catalase (CAT) levels. Furthermore, Hooshmand et al. (2021) inquired about the impact of *Morus nigra* extract (source of anthocyanins) against diethyl nitrosamine‐induced liver cancer in male Sprague–Dawley rats by providing 400 mg/kg extract for 16 weeks. It has been evident that the extract inhibited cell viability by reducing Wnt4 and β‐catenin expression while not influencing the expression of PI3K, Akt, and PTEN (Figure 2).

## 7 Colorectal Cancer

Colorectal cancer is one of the leading causes of mortality worldwide, with approximately 1.8 million reported cases and ~ 0.9 million demises (Li et al. 2024). Genetic modifications in various pathways, including Wnt/β‐catenin, RAS/RAF/MEK/ERK, PI3K/AKT, and TGF‐β, are the root causes in the pathogenesis of colorectal cancer (Oh and Cho 2024). Various clinical, preclinical, and rodent studies have been conducted to ameliorate the elevating burden of colorectal cancer. Researchers have used anthocyanins in multiple antiproliferative trials owing to their cytotoxic and antimetastatic nature. Tumor suppressor genes are significantly mutated by the abnormal microRNA expression involved in the development of colorectal cancer. Previously, Guo et al. (2020) conducted a study to investigate the cytotoxic potential of black raspberry anthocyanins against azoxymethane (AOM)/dextran sulfate sodium‐provoked colorectal cancer in mice as well as in human colorectal cancer cell lines. Significant attenuation in microRNAs (miR‐483‐3p) and upregulation of Dickkopf 3 (DKK3) expression were observed in both models by the consumption of black raspberry anthocyanins. Moreover, the antitumor potential of protein acetylation in black raspberry anthocyanins was investigated against colorectal cancer SW480 and Caco‐2. The results revealed that anthocyanins have significantly influenced acetylation levels by slowing down the expression of *sirtuin1*, *Bcl‐2*, *cyclin D1*, *c‐myc*, and *NLRP3*, as well as modulated *MOF*, *EP300*, NF‐κB, and *Bax* expression, which consequently suppressed the proliferation of colorectal cancer (Chen et al. 2023).

The antiproliferative mechanism of anthocyanins glycosylation on mouse colon cancer (MC38) was evaluated by supplementing bilberry anthocyanins extract (500 μM), and it was shown that the treatment caused mitochondrial deterioration (~48%) and suppressed tumor proliferation (55%) by elevating caspase‐3 and caspase‐9 activity. Additionally, energy metabolism was lowered by modulating GLUT1 inhibitors (Jing et al. 2020). Furthermore, do Nascimento et al. (2023) determined the protective mechanism of jaboticaba peel (enriched with anthocyanins) on inflammation‐induced colorectal cancer in mice by the enrichment of 5% freeze‐dried jaboticaba peel in mice diet for 114 days. The outcomes depicted that adenocarcinoma was alleviated alongside proinflammatory markers, that is, interleukin‐1β, cyclooxygenase‐2, NF‐κB, and inducible nitric oxide synthase, thereby attenuating colorectal cancer. *Aornia melanocarpa Elliot*, a potential source of anthocyanins, has been used to assess its therapeutic impact on the propagation of colorectal cancer (Caco‐2). Significant amelioration in the propagation of Caco‐2 cell lines has been observed which is due to the attenuation of COX‐2, MUC2, IL‐6, IL‐17, TNF‐α, IFN‐γ, MPO, glutaminase (GLS), p‐mTOR, and p‐4EBP1 expression (Yu et al. 2021).

**FIGURE 3:**
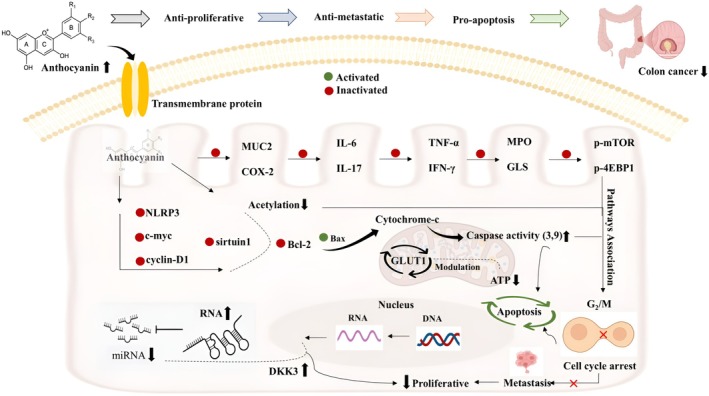
Anthocyanins and their protective effect against colorectal cancer.

A randomized study determined the antimutagenic effect of anthocyanins supplementation in colorectal carcinoma patients (*n* = 35) of 18–85 years by providing 1 g of anthocyanins orally daily for 4–6 weeks. Non‐significant variations in HOMA index, CRP, adiponectin, leptin, IGF‐1, IL‐10, IL‐6, and TNF‐α levels were observed (Macis et al. 2023). Similarly, Geyik et al. (2023) showed that cyanidin‐3‐O‐glucoside chloride, an anthocyanin found in purple and blue wheat brans, positively impacted the amelioration of colorectal cancer proliferation. Hamedi and Koosha (2020) loaded anthocyanins in halloysite nanotubes, which demonstrated improved bioavailability of anthocyanins, thereby modulating the antimutagenic potential of anthocyanins (reduced 2 times) against HT‐29 colon cancer cell lines. Similarly, de Aguiar Cipriano et al. (2022) revealed the antiproliferative mechanism of *Ipomoea batatas* 's anthocyanins against Caco‐2 cell lines. Moreover, Schmutz et al. (2023) used anthocyanins‐enriched blackberry, bilberry, black currant, and elderberry extracts to determine the antimetastatic effect against murine CT26 cell lines. They concluded that blackberry extract showed cytotoxic activity at a specific concentration of 100–200 μg/mL. However, DNA was not affected by blackberry extract. β‐glucosidase‐producing Lactobacillus improved the bioavailability of anthocyanins, which significantly inhibited cancer cell migration and invasion of HT‐29 cells by attenuating COX‐2, iNOS, IL‐6, and IL‐10 (Sirilun et al. 2022). Simas Frauches et al. (2021) also revealed the antiproliferative potential of anthocyanins‐rich *Myrciaria jaboticaba* for HT‐29 cells by arresting the G2/M growth phase (Figure 3).

## 8 Lung Cancer

Lung cancer is the most prevailing cancer, which affects a significant number of individuals worldwide. An estimated 0.226 new cases of lung cancer were recorded by the American Cancer Society with an estimated 0.124 million deaths annually. Food choices, lifestyle practices (exercise, alcoholism, and smoking), environmental contaminations, and heavy metals intake are the root causes of the pathogenesis of lung cancer (Bhat et al. 2024). Recent researchers are linking diet to combating the proliferation of cancers and improving individuals' life expectancy. Anthocyanins have evidenced the cancer suppressive potential against various types of cancers, that is, lung cancer. Zhang et al. (2022) conducted a study to evaluate the inhibitory potential of dietary anthocyanins against lung cancer. It revealed a dose–response relationship as the consumption of anthocyanins increased and the progression of lung cancer declined. Later, Luo et al. (2024) proposed an in vivo study among mice to assess the suppressive mechanism of anthocyanins against lung cancer by enriching the mice's diet with 0.5% anthocyanins. The results showed that oxidative phosphorylation and lipolysis in lung tissues had been significantly attenuated in anthocyanin‐fed mice compared to control mice, demonstrating the lung cancer ameliorating effect. Similarly, Tikhonova et al. (2024) also confirmed the anti‐metastatic effect of anthocyanins against lung cancer by supplementing grains enriched with anthocyanins to mice for 16 weeks. It has been depicted that anthocyanin improved IL‐9 and eotaxin serum levels, whereas it reduced IL‐6/LIF and tumor‐associated M2 macrophage marker arginase 1 gene. Other studies supporting the beneficial role of anthocyanins against lung cancer are described in Table 1.

**TABLE 1:** Anti‐proliferative mechanism of anthocyanins on lung cancer.

Study type	Lung cell	Extract/compound	Dose	Outcomes	References
In vitro	A549	*Vitis coignetiae Pulliat*	200 μg/mL	Reduced levels of cyclin D1, C‐myc, COX‐2, VEGF, and MMP2/9	Lu et al. (2017)
In vitro	A549	*Morus alba* L.	25, 50, and 100 μM	Attenuated NF‐κB, C‐fos, and MMP2	Alsharairi (2022)
In vitro	A549 & H1299	Cyanidin‐3‐glucoside	5, 10, 20, 40, & 80 μM	Lowered P13K/Akt/mTOR and TP53l3	Chen et al. (2021)
In vivo *&* In vitro	A549, H441 & SK‐MES‐1	Delphinidin	5–100 μM (in vitro) & 1, 2 mg PC/kg body weight (in vivo)	Reduced Bcl‐2, PCNA, cyclin D1, VEGFA, EGFR, Akt/PI3K/MAPK while increased Bax and caspase3 & 9	Pal et al. (2013)
In vitro *&* in vivo	A549 & H1299	Bilberry and blueberry	3.125 μM to 12.5 μM in in vitro study while 1.5 mg PC/kg body weight in in vivo study	Suppressed NF‐κB, Notch, Wnt/β‐catenin, cyclin D1/B1, VEGF, Bcl‐2, MMP‐9, and cyclin D1/B1	Kausar et al. (2012)
In vitro	BEAS‐2B	Haskap berry	1–200 μg/mL	Ameliorated NNKOAc stimulated DNA damage & reactive oxygen species, elevated DNA repairing	Amararathna et al. (2020)

## 9 Ovarian Cancer

Ovarian cancer is ranked 5th on the scale of malignancy throughout the world, with epithelial cancer being the most prevalent among others. The American Cancer Society reported ~19,680 ovarian cancer cases among US women, with an estimated 12,740 women dying in 2024. Lifestyle, diet, genetic variations (BRCA1 & BRCA2 genes mutation), and infections (human papillomavirus) are significant etiologies in aggravating the pathogenesis of ovarian cancer (Ullah et al. 2024). Bioactive compounds holding antioxidant and anticancer properties, such as saponins, anthocyanins, and other flavonoids, have gained importance in tumor cell management. The impact of *Kaempferia parviflora* anthocyanins supplementation was studied in the development and proliferation of human ovarian cancer, and it was found that anthocyanin extracts ameliorated the propagation of ES‐2 and OV‐90 cells by apoptosis induction and growth inhibition. Moreover, anthocyanin extracts reduced mitochondrial membrane permeability, along with calcium accumulation and reactive oxygen species (ROS) overproduction in ES‐2 and OV‐90 cells (Kim, Kim, et al. 2022; Kim, Park, et al. 2022).

Previously, Pieńkowska et al. (2021) investigated the potential of Delphinidin, an anthocyanin in red wine and berries, against ovarian cancer cell lines (PEO1 & SKOV3). The results concluded that Delphinidin sensitized the targeted cell lines to 3‐Bromopyruvic acid by alleviating ATP and ROS levels and improving glutathione levels. However, no significant variations in the viability of MRC‐5 fibroblasts were observed up to the concentration of 100 μM. Furthermore, Liu et al. (2023) conducted a cohort study among 700 diagnosed ovarian cancer individuals to determine the effect of colored fruits and vegetables in improving colorectal cancer prognosis. It was depicted that green and red/purple‐colored fruits and vegetables, the main constituents of anthocyanins, attenuated the growth and progression of colorectal cancer.

*Ocimum basilicum* revealed cytotoxicity against the ovarian SKOV‐3 cell lines with IC50 values of 0.9 ± 0.11 mg/mL. Anthocyanin is the principal compound in *Ocimum basilicum* , and it holds cytotoxic potential against ovarian carcinoma (Hanachi et al. 2021). Kohut et al. (2022) investigated the impact of blackcurrants and chokeberry extracts against HGL5 (human ovarian granulosa cells) and OVCAR‐3 (ovarian cancer) cell lines by supplementing 10–100 μg/mL for 24 h. The elevation of HGL5 and OVCAR‐3 viability was observed by 10 μg/mL blackcurrant extract and 10, 20, 50, and 100 μg/mL chokeberry extract, while their viability was suppressed by blackcurrant (20 & 50 μg/mL) and chokeberry extract (100 μg/mL) by releasing 17β‐estradiol and lowering SOD levels. Similarly, Haq et al. (2020) demonstrated that anthocyanins extracted from Romina strawberries suppressed the proliferation of ovarian cancer by decreasing cell viability and depicting IC30, IC50, and IC70 of 13.67, 10.22, and 6.77 μg/mL, respectively.

## 10 Prostate Cancer

Prostate cancer has a significant impact on the survival of individuals, predominantly affecting the normal physiology of men, which leads them to death. Prostate cancer statistics recorded ~0.3 million new cases among US men in the particular year 2024, which accounts for 35,000 demises among men. Mutations in specific signaling pathways such as androgen receptors, PI3K, Ras/Raf/MEK/ERK, and retinoblastoma are dominant during the development of prostate cancer (Mottaghipisheh et al. 2022). The positive effect of anthocyanins on prostate cancer is proved in various studies, which are elaborated here in detail. Li, Mu, et al. (2021), Li, Zhao, et al. (2021), Li, Mi, et al. (2021) aimed to investigate the underlying mechanism of *Lycium ruthenicum* Murray isolated anthocyanins on prostate cancer (DU‐145) by supplementing 0, 100, 200, and 400 μg/mL. It has been evident that anthocyanins attenuated cell proliferation and mitochondrial membrane permeability, which is triggered by regulating Bax and Bcl‐2 expression. Moreover, the activation of PTEN and PI3K/Akt‐stimulated caspase‐3 pathways was observed, consequently ameliorating tumor cell progression. Similarly, Jongsomchai et al. (2020) employed rice bran‐enriched anthocyanins constituting cyanidin 3‐glucosides to evaluate the antimetastatic activity on human prostate cancer (PC3) cell lines and observed suppression of epithelial‐mesenchymal transition of PC3 cells. Moreover, cyanidin 3‐glucosides downregulated the expression of Smad/Snail signaling pathways while modulating the E‐cadherin protein. Further studies linking anthocyanins in reducing the prevalence of prostate cancer are described in Table 2.

**TABLE 2:** Anti‐proliferative mechanism of anthocyanins against prostate cancer.

Study	Extract/compound	Dose	Results	References
In vitro	Blue Corn	—	↓cell viability, promoted apoptosis	Herrera‐Sotero et al. (2020)
In vivo	Purple corn	2, 10, and 50 mg/kg body weight for 4 weeks	↓cell migration & proliferation, ↑PI3K/Akt, ↓androgen receptors	Kim, Kim, et al. (2022) and Kim, Park, et al. (2022)
In vitro	*Aronia melanocarpa*	100 mg/kg body weight for 6 weeks	↓prostate enlargement, ↓dihydrotestosterone & 5α‐reductase	Kim, Paramanantham, et al. (2020) and Kim, Jegal, et al. (2020)
In vivo	*Vaccinium myrtillus* berry	14.15 & 113.2 μg	↑apoptosis, ↓tumor cell growth and invasion	Del Bubba et al. (2021)
In vitro	*Vitis vinifera* L.	14.46 mg/100 g malvidin‐3‐O‐glycoside	↓cell growth & progression	Cruz et al. (2023)
In vivo	Red Sorghum	—	↓cell viability, ↑NF‐κB, CREB, SOX, and CDH1 activities	Mora et al. (2024)
In vivo *and* in vitro	Purple Rice	1% for in vivo, and 0.2% for in vitro	↓tumor growth & proliferation, ↓androgen receptors, cyclin D1, CDK4, & fatty acid synthase	Yeewa et al. (2020)
In vivo	Brazilian Berry	125, 250, and 500 μg/mL	↓cell progression, ↑cytotoxicity, ↑NF‐κB, ↑pro‐inflammatory (COX‐2), immunostimulatory & immunosuppressive genes (cytokine‐cytokine interactions)	Kido et al. (2022)
In vitro	Black mulberry	100, 200, 300, and 400 mg/kg body weight for 6 weeks	↑TNFα, PEG2, oxidative stress, and prostate weight in positive manner	Abd El‐Rahman and Mohamed Amin Tolba (2020)

## 11 Gastric Cancer

Gastric cancer accounts for significant mortality rates and is characterized by genetic mutations (tumor suppressor gene modifications), the imbalance between cell proliferation and apoptosis, enhanced inflammation, compromised lifestyle, and dietary practices (Dharmawansa et al. 2020). The American Cancer Society estimated that ~26,890 newly diagnosed cases are reported in 2024, out of which 10,880 patients have lost their lives. Managing gastric cancer through a plant‐based diet and compounds has attained significant importance. Therefore, anthocyanins have been used to cure gastric cancer, and their relevant studies are described here comprehensively. Anthocyanins and procyanidins are found in abundant amounts in red, which were identified and separated by Wang et al. (2024). They used anthocyanins and procyanidins collectively to evaluate their suppressive mechanism on the growth of gastric cancer. The results revealed the inhibition of MKN‐28 cell viability by the synergistic activity of anthocyanins and procyanidins. The underlying mechanism was associated with promoting apoptotic pathways, Bcl‐2/Bax, cell growth phase, and HK2 protein suppression. Furthermore, de Mejia et al. (2021) concluded that anthocyanins have ameliorated the pathogenesis of gastric cancer by reducing inflammation, reactive oxygen, and nitrogen species (ROS and RNS), thereby attenuating oxidative stress.

The molecular pathway of cyanidin‐3‐O‐glucoside against human gastric cancer (MKN‐45) cell lines was studied, which showed downregulation of mitochondrial membrane potential, G2/M phase suppression, and apoptosis activation. Moreover, the activation of E‐cadherin protein, ROS, MAPK, NF‐κB, and STAT3 alongside inhibited tumor cell migration was observed by cyanidin‐3‐O‐glucoside (Sun et al. 2023). *Vaccinium myrtillus* L. is consumed as a fruit source that is enriched with anthocyanins. Karakaş et al. (2022) extracted anthocyanins from the methanol extract of *Vaccinium myrtillus* L. and investigated the anticancer potential against gastric cancer (AGS cell lines). Obviously, the supplementation of the methanol extract of *Vaccinium myrtillus* L. (2 mg/mL) decreased gastric cell viability. Similarly, Calderón‐Reyes et al. (2020) investigated the anti‐proliferative mechanism of anthocyanins‐enriched calafate fruits against stomach cancer. They concluded that the anthocyanin‐rich extract alleviated ~70% of tumor cell growth, migration, and invasion compared to the crude extract. The main compounds of anthocyanins in calafate fruits are delphinidin, malvidin, cyanidin, peonidin, and petunidin, which enhance the antimetastatic activity of anthocyanins.

Ribera‐Fonseca et al. (2020) isolated anthocyanins from the leaves of blueberries and found a similar effect on human gastric cancer cell lines. The protective effect of blueberry anthocyanins against human gastric cancer (BGC‐823) and implanted tumors in nude mice was evaluated by supplementing 0, 50, and 100 μg/mL of anthocyanins. The results revealed that elevated levels of anthocyanins reduced the propagation of tumor cells and promoted apoptosis. Additionally, B‐cell lymphoma 2 protein and tumor growth were suppressed, while *Bcl‐2* and p‐c‐Jun N‐terminal kinase protein were improved by anthocyanins (Huang et al. 2023). Similar behavior was observed when anthocyanins from *Vitis coignetiae pulliat* were employed by Park, Lee, et al. (2021) and Park, Kim, et al. (2021) to evaluate their role in gastric cancer proliferation. It has been found that anthocyanins induced apoptosis by elevating caspase‐3, caspase‐9, and ROS production and lowering XIAP, Bcl‐2, and anti‐apoptotic protein. Moreover, Santos et al. (2024) also observed that *Açaí* extract had alleviated the proliferation and metastatic activity of APG01 gastric cell lines.

## 12 Renal Cancer

Renal cancer is a malignant tumor that accounts for ~90% of all renal abnormalities and is characterized by excessive alcohol, tobacco, and cigarette consumption along with hypertension, chronic renal failure, and other deleterious chemical exposure (Pandey and Syed 2024). Cirillo et al. (2024) estimated that globally ~0.4 million new cases of renal cancer are recorded annually with 0.175 million deaths. Low‐ and middle‐income countries are at a high risk of developing renal cancer due to the inflation rate and the unavailability of quality food and healthcare facilities. Traditional healthcare systems rely on medicinal plants and their prepared decoctions to reduce and manage the disease burden among communities (Fayiah et al. 2024). This concept is continuously increasing due to cheaper treatment with limited adverse health consequences (Sundarrajan and Bhagtaney 2024). Anthocyanins are one of the bioactive compounds extracted from medicinal plants and possess anticancer potential. Various trials regarding the anticancer effect of anthocyanins against renal cell carcinoma are comprehensively presented here. Xu et al. (2023) conducted a prospective study to determine the association between anthocyanin consumption and the risk of renal carcinoma among 409 renal cancer patients. An inverse relationship has been revealed that the proliferation and growth of renal cell carcinoma significantly declined with the intake of anthocyanins. The in vitro protective mechanism of cyanidin on the proliferation of 786–0 (renal cell carcinoma) and ACHN (papillary renal cell carcinoma) was evaluated by providing 0.5 to 15 μM cyanidin for one to three days. The results showed that cyanidin inhibited the growth and migration of tumor cells by promoting apoptosis, ERG1, and cell growth phase (G1/M) suppression as well as by attenuating SEPW1, HIF2A, Bcl‐2, E‐cadherin, caspase‐3, and ROS production (Liu et al. 2018). Furthermore, Wei et al. (2018) proposed in vivo and in vitro studies to investigate the underlying molecular mechanism of anthocyanins on apoptosis metabolism and revealed that anthocyanins intake alleviated ROS, cleaved caspase‐3, Bax/Bcl‐2, MAPK, and ERK1/2 expression.

## 13 Oral Cancer

Oral squamous cell carcinoma is the most common and rapidly growing malignant tumor of the oral cavity, affecting approximately half a million newly diagnosed cases per year. Multiple contributors such as unhygienic oral health, poor lifestyle behaviors (smoking, tobacco, and alcohol consumption), as well as oral microbial infections make significant contributions to the development of oral cancer among individuals (Belibasakis et al. 2024). The positive association of anthocyanins with oral squamous cell carcinoma is evidenced by numerous studies, which are shown here. For instance, Mauramo et al. (2021) conducted an in vivo and in vitro study to investigate the antimetastatic potential of bilberry powder, constituting anthocyanins, on oral carcinoma (HSC‐3) cells by supplementing 0, 1, 10, and 25 mg/mL of bilberry powder. It has been concluded that tumor growth, invasion, viability, and migration of HSC‐3 cell lines were suppressed by bilberry powder. However, it has been shown that the anti‐proliferative potential was improved as the supplementation of bilberry powder increased. Previously, Prakash et al. (2021) and Baba et al. (2017) utilized *Vaccinium corymbosum* L. to evaluate the protective mechanism against SCC131 cancer cell lines in an in vivo and in vitro model. The dietary supplementation of *Vaccinium corymbosum* L. (200 mg/kg) attenuated the growth and proliferation of SCC131 by downregulating the expression of TGF‐β and NF‐κB.

The antiproliferative potential of anthocyanins extracted from *Bridelia retusa* was analyzed against SCC4, SCC9, and SCC25 cell lines by an in vitro trial. Anthocyanins restricted the S‐G2/M and G0/G1 cell growth phases and stimulated the apoptosis process to reduce squamous cell carcinoma propagation. Moreover, caspase‐3 levels have improved from 1.5 to 3 times with the IC50 and IC80 values of 134% and 267%, respectively, compared to a control group with 89% inhibition activity (Madanakumar et al. 2018). Similarly, the intake of ~250 μg/mL of anthocyanins from *Vaccinium corymbosum* for two consecutive days suppressed the viability of oral squamous cell carcinoma by modulating caspase‐1, NLRP3, and IL‐1β expression (Yue et al. 2019). Later, Nedungadi et al. (2022) inquired about the potential of black raspberry administration in modulating the glucocorticoid system to prevent oral carcinoma (CAL27). The results depicted that the enrichment of 5% black raspberry into the diet of C57Bl/6 mice and F344 rats upregulated the activity of the glucocorticoid system, which ultimately suppressed the HSD11B2 enzyme to reduce the burden of CAL27 cell lines. The cytotoxic activity of *Vaccinium macrocarpon* was evaluated against oral cancer KB cell lines and demonstrated the suppressive effect by reducing cell proliferation and invasion (Ankola et al. 2020).

## 14 Cervical Cancer

Cervical cancer is one of the malignant tumors among women in both developed and developing countries. The mortality rates are increasing due to cervical cancer, which in turn results in disturbing the normal reproductive physiology in females. The World Health Organization revealed that the prevalence of cervical cancer in 2022 was ~0.66 million, out of which ~0.349 million deaths were recorded (Wu et al. 2024). Various infections, including human papillomavirus (HPV), human immunodeficiency virus (HIV), misuse of oral contraceptives, multiple and repeated pregnancies, and limited fruit and vegetable intake, contribute to enhancing the pathogenesis of cervical cancer (Yang et al. 2024). Anthocyanins ameliorate the occurrence of cervical cancer by downregulating the tumor promoter genes, and their evidence is presented in Table 3.

**TABLE 3:** Cervical cancer attenuating potential of anthocyanins.

Study	Compound	Dose	Results	References
In vitro study against Hela cell lines	Cyanidin‐3‐O‐glucoside	400 μg/mL	Inhibited tumor growth, apoptosis induction, ↓cyclin D1, Bcl‐2, and PI3K/Akt/mTOR pathway, ↑caspase‐3, p53, and TIMP‐1	Li, Mu, et al. (2021), Li, Zhao, et al. (2021), and Li, Mi, et al. (2021)
In vitro investigation on the metastasis of Hela cells	Cyanidin	1.89 μg/mL	Alleviated cell proliferation, ROS production, and peroxides levels	Drețcanu et al. (2021) and Diaconeasa et al. (2017)
Antiproliferative potential against Hela & Hela S3 cell lines	Delphinidin	25–200 μM for a day	↓c‐junction, ↓cell invasion & propagation, ↑apoptosis, ↑autophagosome formation	Teniente et al. (2023) and Tsuyuki et al. (2012)
In vitro evaluation on Hela cell lines	*Aronia melanocarpa* (Michx.) Elliot	—	Higher cytotoxicity (IC_50_ = 95 μg/mL) by ethanol extract	Gürer and Altıntaş (2024)
In vitro	Anthocyanins	100 & 200 μg/mL for two consecutive days	↑CFP/YFP, suppressed tumor growth, induced apoptosis	Park, Lee, et al. (2021), Park, Kim, et al. (2021), and Vishnu et al. (2019)
In vitro	Cyanidin‐3‐O‐glucoside	0, 4, 8, 12, 16, and 20 μM for 48 h	↑ROS levels, ↓mitochondrial membrane potential, ↑production of PGC‐1α	Liu et al. (2024)
In vivo	Cyanidin‐3‐O‐glucoside	40 mg/kg body weight	↓Bcl‐2, ↑Bax & caspase‐3 expression, modulation of PI3K/AKT/mTOR signaling pathway	Li, Mu, et al. (2021), Li, Zhao, et al. (2021), and Li, Mi, et al. (2021)

## 15 Uterine Cancer

Uterine cancer, the sixth most common type of cancer, is prevalent among females and accounted for approximately 67,880 newly diagnosed incidences in 2024. The National Cancer Institute recorded 13,250 deaths in the United States in the respective year. Genetic (variation in BRCA1 and BRCA2 expression), hormonal (long‐term estrogen exposure), obesity, diabetes mellitus, environmental, dietary, and lifestyle practices promote the pathogenesis of uterine cancer (Lakkis et al. 2024; Meena et al. 2024). Therefore, its management is crucial to reduce the incidences of uterine cancer as well as its associated abnormalities. The anticancer effects of anthocyanins are proven against various types of cancer, including uterine cancer, and are designated here. Yang et al. (2022) conducted a study investigating the impact of anthocyanin cyanidin‐3‐*O*‐glucoside on the incidence of cadmium‐induced uterine epithelium in mice. It has been observed that cyanidin‐3‐*O*‐glucoside alleviated the metastasis of uterine epithelium owing to its anti‐estrogenic potential. This effect was shown by suppressing estrogen‐associated genes, modulating Kif4 expression, and improving epithelial progesterone receptor expression. Anthocyanins also ameliorated the invasion of uterine cancer by lowering the expression of extracellular matrix production and reducing inflammatory markers (Ciebiera et al. 2020).

## 16 Future Perspectives

Even though anthocyanins have anticancer potential, further investigations are required to maximize their stability, bioavailability, and therapeutic attributes. Anthocyanins are rapidly metabolized in the gastrointestinal tract, so their bioavailability is a barrier in utilizing anthocyanins as anti‐cancer agents. Future research should focus on the target delivery systems and formulations to improve the bio‐accessibility of anthocyanins. Furthermore, underlying anticancer molecular and epigenetic pathways associated with anthocyanins are required to investigate, which could further illustrate their clinical significance. Standardized treatment recommendations should be established by conducting clinical studies to better examine the pharmacokinetics, pharmacodynamics, and ideal dose regimens of anthocyanins against various cancer types. In this regard, artificial intelligence could be beneficial in early cancer detection, optimizing treatment regimens, and developing novel drugs. Anthocyanins alone and in combination with other compounds and drugs should be evaluated to discover novel and innovative anticancer strategies. Moreover, the development of functional foods and nutraceuticals of anthocyanins could facilitate individuals with therapeutic agents after the validation of preclinical and clinical trials.

## 17 Conclusions

Anthocyanins are the bioactive compounds of numerous fruits and vegetables that hold substantial anticancer potential against breast, hepatocellular, stomach, kidney, uterine, and colorectal cancers. Research has demonstrated that anthocyanins extracted from purple sweet potatoes, *Lycium ruthenicum* Murray, and *Vitis coignetiae* Pulliat inhibit cancer cells' ability to migrate, invade, and proliferate. The bioavailability of anthocyanins significantly impacts the therapeutic potential, which is improved by the addition of other compounds or by formulating nano‐emulsions and nanoparticles for target delivery. Clinical studies evidenced the therapeutic potential of anthocyanins by modulating various signaling pathways such as NF‐κB, PI3K/Akt, MAPK, and apoptotic cascades to attenuate the metastasis of abnormal and affected tumor cells. Moreover, anthocyanins regulate the activity of tumor suppressor genes, that is, *TP53*, *RB1*, *and BRCA1*, and suppress tumor‐promoting genes such as *Bcl‐2*, *MYC*, and *HER2*, consequently ameliorating cancer proliferation. Moreover, as evident in colorectal and breast cancer trials, anthocyanins have demonstrated dose‐dependent anticancer action through oxidative stress regulation, cell cycle arrest, and mitochondrial malfunction.

## Author Contributions

**Muhammad Maaz:** conceptualization (equal), writing – original draft (equal). **Muhammad Tauseef Sultan:** conceptualization (equal), writing – original draft (equal). **Ahmad Mujtaba Noman:** resources (equal), writing – original draft (equal). **Shehnshah Zafar:** investigation (equal), writing – review and editing (equal). **Naima Tariq:** software (equal), writing – review and editing (equal). **Muzzamal Hussain:** supervision (equal), writing – review and editing (equal). **Muhammad Imran:** data curation (equal), resources (equal), validation (equal). **Ahmed Mujtaba:** validation (equal), visualization (equal). **Tadesse Fenta Yehuala:** data curation (equal), formal analysis (equal), resources (equal), supervision (equal), writing – review and editing (equal). **Ehab M. Mostafa:** data curation (equal), methodology (equal). **Samy Selim:** data curation (equal), visualization (equal). **Soad K. Al Jaouni:** writing – review and editing (equal). **Suliman A. Alsagaby:** data curation (equal), investigation (equal). **Waleed Al Abdulmonem:** writing – review and editing (equal).

## Conflicts of Interest

The authors declare no conflicts of interest.

## Data Availability

The data that support the findings of this study are available on request from the corresponding author.

## References

[fsn370232-bib-0001] Abd El‐Rahman, A. M. M. , and A. Mohamed Amin Tolba . 2020. “Study the Effect of Natural Antioxidants in Mulberry on Prostate Cancer in Male Wister Albino.” مجلة الاقتصاد المنزلي 36, no. 1: 71–92.

[fsn370232-bib-0002] Al‐Khayri, J. M. , W. Asghar , A. Akhtar , et al. 2022. “Anthocyanin Delivery Systems: A Critical Review of Recent Research Findings.” Applied Sciences 12, no. 23: 12347. 10.3390/app122312347.

[fsn370232-bib-0003] Alsharairi, N. A. 2022. “Insights Into the Mechanisms of Action of Proanthocyanidins and Anthocyanins in the Treatment of Nicotine‐Induced Non‐Small Cell Lung Cancer.” International Journal of Molecular Sciences 23, no. 14: 7905. 35887251 10.3390/ijms23147905 PMC9316101

[fsn370232-bib-0004] Amararathna, M. , D. W. Hoskin , and H. V. Rupasinghe . 2020. “Anthocyanin‐Rich Haskap (*Lonicera Caerulea* L.) Berry Extracts Reduce Nitrosamine‐Induced DNA Damage in Human Normal Lung Epithelial Cells *In Vitro* .” Food and Chemical Toxicology 141: 111404. 32413456 10.1016/j.fct.2020.111404

[fsn370232-bib-0005] Amararathna, M. , D. W. Hoskin , and H. V. Rupasinghe . 2022. “Anthocyanin Encapsulated Nanoparticles as a Pulmonary Delivery System.” Oxidative Medicine and Cellular Longevity 2022, no. 1: 1422929. 36124088 10.1155/2022/1422929 PMC9482540

[fsn370232-bib-0006] Ankola, A. V. , V. Kumar , S. Thakur , R. Singhal , T. Smitha , and R. Sankeshwari . 2020. “Anticancer and Antiproliferative Efficacy of a Standardized Extract of *Vaccinium macrocarpon* on the Highly Differentiating Oral Cancer KB Cell Line Athwart the Cytotoxicity Evaluation of the Same on the Normal Fibroblast L929 Cell Line.” Journal of Oral and Maxillofacial Pathology 24, no. 2: 258–265. 10.4103/jomfp.JOMFP_129_20. 33456234 PMC7802834

[fsn370232-bib-0007] Aqil, F. , R. Munagala , A. K. Agrawal , et al. 2021. “Anthocyanidins Inhibit Growth and Chemosensitize Triple‐Negative Breast Cancer via the NF‐κB Signaling Pathway.” Cancers 13, no. 24: 6248. 34944868 10.3390/cancers13246248 PMC8699375

[fsn370232-bib-0008] Arango‐Varela, S. S. , D. Torres‐Camargo , C. Reyes‐Dieck , M. B. Zapata‐Londoño , and M. E. Maldonado‐Celis . 2021. “Aqueous Extract of Andean Berry Induces Apoptosis in Human Colon Cancer Cells Without Mitochondrial Damage.” Journal of Berry Research 11, no. 3: 377–393.

[fsn370232-bib-0009] Awad, M. G. , N. A. Hanafy , R. A. Ali , D. D. Abd El‐Monem , S. H. El‐Shafiey , and M. A. El‐Magd . 2024. “Exploring the Therapeutic Applications of Nano‐Therapy of Encapsulated Cisplatin and Anthocyanin‐Loaded Multiwalled Carbon Nanotubes Coated With Chitosan‐Conjugated Folic Acid in Targeting Breast and Liver Cancers.” International Journal of Biological Macromolecules 280, no. Pt 2: 135854. 10.1016/j.ijbiomac.2024.135854. 39307483

[fsn370232-bib-0010] Baba, A. B. , R. Nivetha , I. Chattopadhyay , and S. Nagini . 2017. “Blueberry and Malvidin Inhibit Cell Cycle Progression and Induce Mitochondrial‐Mediated Apoptosis by Abrogating the JAK/STAT‐3 Signalling Pathway.” Food and Chemical Toxicology 109: 534–543. 28974439 10.1016/j.fct.2017.09.054

[fsn370232-bib-0011] Belibasakis, G. N. , C. J. Seneviratne , R. D. Jayasinghe , P. T. D. Vo , N. Bostanci , and Y. Choi . 2024. “Bacteriome and Mycobiome Dysbiosis in Oral Mucosal Dysplasia and Oral Cancer.” Periodontology 2000 96, no. 1: 95–111. 10.1111/prd.12558. 38501658 PMC11579824

[fsn370232-bib-0012] Bhat, A. A. , E. Moglad , P. Bansal , et al. 2024. “Pollutants to Pathogens: The Role of Heavy Metals in Modulating TGF‐β Signaling and Lung Cancer Risk.” Pathology, Research and Practice 256: 155260. 38493726 10.1016/j.prp.2024.155260

[fsn370232-bib-0013] Bhol, N. K. , M. M. Bhanjadeo , A. K. Singh , et al. 2024. “The Interplay Between Cytokines, Inflammation, and Antioxidants: Mechanistic Insights and Therapeutic Potentials of Various Antioxidants and Anti‐Cytokine Compounds.” Biomedicine & Pharmacotherapy 178: 117177. 10.1016/j.biopha.2024.117177. 39053423

[fsn370232-bib-0014] Bosch, T. C. , M. Wigley , B. Colomina , et al. 2024. “The Potential Importance of the Built‐Environment Microbiome and Its Impact on Human Health.” Proceedings of the National Academy of Sciences 121, no. 20: e2313971121. 10.1073/pnas.2313971121 PMC11098107 38662573

[fsn370232-bib-0015] Bracone, F. , A. De Curtis , A. Di Castelnuovo , et al. 2021. “Skin Toxicity Following Radiotherapy in Patients With Breast Carcinoma: Is Anthocyanin Supplementation Beneficial?” Clinical Nutrition 40, no. 4: 2068–2077. 33051045 10.1016/j.clnu.2020.09.030

[fsn370232-bib-0016] Calderón‐Reyes, C. , R. S. Pezoa , P. Leal , et al. 2020. “Anthocyanin‐Rich Extracts of Calafate (*Berberis microphylla* g. Forst.) Fruits Decrease *In Vitro* Viability and Migration of Human Gastric and Gallbladder Cancer Cell Lines.” Journal of Soil Science and Plant Nutrition 20, no. 4: 1891–1903. 10.1007/s42729-020-00260-8.

[fsn370232-bib-0017] Catacutan, M. K. , T. Y. Kim , and S. Lee . 2024. “Selective Cytotoxicity of Anthocyanins on Breast Cancer Cells.”

[fsn370232-bib-0018] Charron, C. S. , A. C. Kurilich , B. A. Clevidence , et al. 2009. “Bioavailability of Anthocyanins From Purple Carrot Juice: Effects of Acylation and Plant Matrix.” Journal of Agricultural and Food Chemistry 57, no. 4: 1226–1230. 10.1021/jf802988s. 19166298

[fsn370232-bib-0019] Chen, L. , M. Li , H. Zhou , et al. 2023. “Sirtuin1 (SIRT1) is Involved in the Anticancer Effect of Black Raspberry Anthocyanins in Colorectal Cancer.” European Journal of Nutrition 62, no. 1: 395–406. 10.1007/s00394-022-02989-7. 36056948

[fsn370232-bib-0020] Chen, Q. , F. Rui , W. Ni , and J. Li . 2024. “Research Progress in Epidemiology and Risk Factors of Primary Liver Cancer.” Chinese General Practice 27, no. 06: 637.

[fsn370232-bib-0021] Chen, X. , W. Zhang , and X. Xu . 2021. “Cyanidin‐3‐Glucoside Suppresses the Progression of Lung Adenocarcinoma by Downregulating TP53I3 and Inhibiting PI3K/AKT/mTOR Pathway.” World Journal of Surgical Oncology 19: 1–12. 34362378 10.1186/s12957-021-02339-7 PMC8348822

[fsn370232-bib-0022] Ciebiera, M. , M. Ali , L. Prince , et al. 2020. “The Evolving Role of Natural Compounds in the Medical Treatment of Uterine Fibroids.” Journal of Clinical Medicine 9, no. 5: 1479. 32423112 10.3390/jcm9051479 PMC7290481

[fsn370232-bib-0023] Cirillo, L. , S. Innocenti , and F. Becherucci . 2024. “Global Epidemiology of Kidney Cancer.” Nephrology, Dialysis, Transplantation 39, no. 6: 920–928. 10.1093/ndt/gfae036 38341277

[fsn370232-bib-0024] Cruz, M. A. D. A. S. , G. D. F. L. Pascoal , M. E. D. S. Jacintho , et al. 2023. “Antiproliferative and Apoptosis Effects of Hybrid Varieties of *Vitis vinifera* L. Sweet Sapphire and Sweet Surprise on Human Prostate Cancer Cells Using *in Vitro* and in Silico Approaches.” Asian Pacific Journal of Cancer Prevention 24, no. 11: 3673. 38019224 10.31557/APJCP.2023.24.11.3673 PMC10772743

[fsn370232-bib-0025] da Silva, D. T. , F. A. Smaniotto , I. F. Costa , et al. 2021. “Natural Deep Eutectic Solvent (NADES): A Strategy to Improve the Bioavailability of Blueberry Phenolic Compounds in a Ready‐To‐Use Extract.” Food Chemistry 364: 130370. 10.1016/j.foodchem.2021.130370. 34182361

[fsn370232-bib-0026] de Aguiar Cipriano, P. , H. Kim , C. Fang , V. P. Venancio , S. U. Mertens‐Talcott , and S. T. Talcott . 2022. “ *In Vitro* Digestion, Absorption and Biological Activities of Acylated Anthocyanins From Purple Sweet Potatoes (*Ipomoea batatas*).” Food Chemistry 374: 131076. 10.1016/j.foodchem.2021.131076. 34915366

[fsn370232-bib-0027] de Mejia, E. G. , M. Rebollo‐Hernanz , Y. Aguilera , and M. A. Martín‐Cabrejas . 2021. “Role of Anthocyanins in Oxidative Stress and the Prevention of Cancer in the Digestive System.” In Cancer, 265–280. Academic Press.

[fsn370232-bib-0028] Dehelean, C. A. , and I. A. Pînzaru . 2024. “Cooperation to Implement Innovative Methods for the Assessment of Medicinal Plants with Central Roles in Pharmaceutics, Agriculture and Nutrition.”

[fsn370232-bib-0029] Del Bubba, M. , C. Di Serio , L. Renai , et al. 2021. “ *Vaccinium myrtillus* L. Extract and Its Native Polyphenol‐Recombined Mixture Have Anti‐Proliferative and Pro‐Apoptotic Effects on Human Prostate Cancer Cell Lines.” Phytotherapy Research 35, no. 2: 1089–1098. 32929801 10.1002/ptr.6879

[fsn370232-bib-0030] Dharmawansa, K. S. , D. W. Hoskin , and H. V. Rupasinghe . 2020. “Chemopreventive Effect of Dietary Anthocyanins Against Gastrointestinal Cancers: A Review of Recent Advances and Perspectives.” International Journal of Molecular Sciences 21, no. 18: 6555. 10.3390/ijms21186555. 32911639 PMC7554903

[fsn370232-bib-0031] Diaconeasa, Z. , H. Ayvaz , D. Ruginǎ , et al. 2017. “Melanoma Inhibition by Anthocyanins is Associated With the Reduction of Oxidative Stress Biomarkers and Changes in Mitochondrial Membrane Potential.” Plant Foods for Human Nutrition 72: 404–410. 29129015 10.1007/s11130-017-0638-x

[fsn370232-bib-0032] Diniz, D. G. , J. Bento‐Torres , V. O. da Costa , et al. 2024. “The Hidden Dangers of Sedentary Living: Insights Into Molecular, Cellular, and Systemic Mechanisms.” International Journal of Molecular Sciences 25, no. 19: 10757. 10.3390/ijms251910757. 39409085 PMC11476792

[fsn370232-bib-0033] do Nascimento, R. D. P. , J. S. Rizzato , G. Polezi , et al. 2023. “Freeze‐Dried Jaboticaba (*Myrciaria jaboticaba*) Peel Powder, a Rich Source of Anthocyanins and Phenolic Acids, Mitigates Inflammation‐Driven Colorectal Cancer in Mice.” Food Bioscience 53: 102578. 10.1016/j.fbio.2023.102578.

[fsn370232-bib-0034] dos Santos, S. S. , C. M. Paraíso , E. B. Romanini , et al. 2022. “Bioavailability of Blackberry Pomace Microcapsules by Using Different Techniques: An Approach for Yogurt Application.” Innovative Food Science & Emerging Technologies 81: 103111. 10.1016/j.ifset.2022.103111.

[fsn370232-bib-0035] Drețcanu, G. , C. I. Iuhas , and Z. Diaconeasa . 2021. “The Involvement of Natural Polyphenols in the Chemoprevention of Cervical Cancer.” International Journal of Molecular Sciences 22, no. 16: 8812. 34445518 10.3390/ijms22168812 PMC8396230

[fsn370232-bib-0036] Du, J. , L. Liu , H. Fan , et al. 2023. “Anthocyanins Improve Liver Fibrosis in Mice by Regulating the Autophagic Flux Level of Hepatic Stellate Cells by mmu_circ_0000623.” Food Science & Nutrition 11, no. 6: 3002–3018. 10.1002/fsn3.3281. 37324880 PMC10261807

[fsn370232-bib-0037] Eze, F. N. , R. C. Eze , K. E. Okpara , A. E. Adekoya , and H. N. Kalu . 2024. “Design and Development of Locust Bean Gum‐Endowed/Phyllanthus Reticulatus Anthocyanin‐Functionalized Biogenic Gold Nanosystem for Enhanced Antioxidative and Anticancer Chemotherapy.” International Journal of Biological Macromolecules 275: 133687. 38972650 10.1016/j.ijbiomac.2024.133687

[fsn370232-bib-0038] Fan, H. , Y. Ji , Y. Wang , et al. 2022. “Anthocyanins from Lycium Ruthenicum Murray Inhibit HepG2 Cells Growth, Metastasis and Promote Apoptosis and G2/M Phase Cycle Arrest by Activating the AMPK/mTOR Autophagy Pathway.” Evidence‐Based Complementary and Alternative Medicine 2022, no. 1: 9609596. 36619198 10.1155/2022/9609596 PMC9822762

[fsn370232-bib-0039] Fayiah, M. , M. S. Fayiah , S. Saccoh , and M. K. Kallon . 2024. “Value of Herbal Medicine to Sustainable Development.” In Herbal Medicine Phytochemistry: Applications and Trends, 1429–1456. Springer International Publishing.

[fsn370232-bib-0040] Germán‐Soto, B. G. , J. B. Heredia , N. Leyva‐López , et al. 2024. “Bioaccessibility and Antioxidant Capacity of Alkaloids From Microencapsulated Extract of Eggplant (*Solanum melongena* L.) Biomass.” Horticulturae 10, no. 12: 1242. 10.3390/horticulturae10121242.

[fsn370232-bib-0041] Geyik, Ö. G. , Z. H. Tekin‐Cakmak , V. P. Shamanin , et al. 2023. “Effects of Phenolic Compounds of Colored Wheats on Colorectal Cancer Cell Lines.” Quality Assurance & Safety of Crops and Food 15, no. 4: 21–31.

[fsn370232-bib-0042] Gonçalves, A. C. , A. R. Nunes , A. Falcão , G. Alves , and L. R. Silva . 2021. “Dietary Effects of Anthocyanins in Human Health: A Comprehensive Review.” Pharmaceuticals 14, no. 7: 690. 34358116 10.3390/ph14070690 PMC8308553

[fsn370232-bib-0043] Guo, J. , Z. Yang , H. Zhou , et al. 2020. “Upregulation of DKK3 by miR‐483‐3p Plays an Important Role in the Chemoprevention of Colorectal Cancer Mediated by Black Raspberry Anthocyanins.” Molecular Carcinogenesis 59, no. 2: 168–178. 31763724 10.1002/mc.23138

[fsn370232-bib-0044] Guo, L. , Y. Jin , Y. Yang , et al. 2022. “Calcicoptosis Induced by Purple Sweet Potato Anthocyanins Through the Nonosmotic Regulation of the NFAT5/S100A4‐S100A9 Pathway in Acute Lymphoblastic Leukemia.” Chemistry & Biodiversity 19, no. 9: e202200447. 10.1002/cbdv.202200447. 35924786

[fsn370232-bib-0045] Gürer, E. S. , and A. Altıntaş . 2024. “Determination of Cytotoxic Activity of *Aronia melanocarpa* (Michx.) Elliot Fruit Extracts on Breast Cancer (MCF‐7) and Cervical Cancer (HeLa) Cell Lines.” Cumhuriyet Science Journal 45, no. 3: 537–542.

[fsn370232-bib-0046] Hamedi, S. , and M. Koosha . 2020. “Designing a pH‐Responsive Drug Delivery System for the Release of Black‐Carrot Anthocyanins Loaded in Halloysite Nanotubes for Cancer Treatment.” Applied Clay Science 197: 105770.

[fsn370232-bib-0047] Han, F. , P. Yang , H. Wang , I. Fernandes , N. Mateus , and Y. Liu . 2019. “Digestion and Absorption of Red Grape and Wine Anthocyanins Through the Gastrointestinal Tract.” Trends in Food Science & Technology 83: 211–224.

[fsn370232-bib-0048] Hanachi, P. , F. R. Fakhrnezhad , R. Zarringhalami , and I. E. Orhan . 2021. “Cytotoxicity of Ocimum Basilicum and *Impatiens Walleriana* Extracts on AGS and SKOV‐3 Cancer Cell Lines by Flow Cytometry Analysis.” International Journal of Cancer Management 14, no. 3: e102610.

[fsn370232-bib-0049] Haq, R. S. , T. Y. Forbes‐Hernandez , D. Cianciosi , J. Ansari , M. Battino , and F. Giampieri . 2020. “Abstract A14: The Chemopreventive Effects of Natural Compounds in Romina Strawberries in Human Ovarian Cancer Cells.” Clinical Cancer Research 26, no. 13_Supplement: A14.

[fsn370232-bib-0050] Herrera‐Sotero, M. Y. , C. D. Cruz‐Hernández , R. M. Oliart‐Ros , et al. 2020. “Anthocyanins of Blue Corn and Tortilla Arrest Cell Cycle and Induce Apoptosis on Breast and Prostate Cancer Cells.” Nutrition and Cancer 72, no. 5: 768–777. 10.1080/01635581.2019.1654529. 31448633

[fsn370232-bib-0051] Hooshmand, S. , M. R. Mahdinezhad , S. Taraz Jamshidi , et al. 2021. “ *Morus nigra* L. Extract Prolongs Survival of Rats With Hepatocellular Carcinoma.” Phytotherapy Research 35, no. 6: 3365–3376. 33624311 10.1002/ptr.7056

[fsn370232-bib-0052] Hu, J. , Q. Qi , Y. Zhu , et al. 2023. “Unveiling the Anticancer, Antimicrobial, Antioxidative Properties, and UPLC‐ESI‐QTOF‐MS/GC–MS Metabolite Profile of the Lipophilic Extract of Siam Weed ( *Chromolaena odorata* ).” Arabian Journal of Chemistry 16, no. 7: 104834.

[fsn370232-bib-0053] Huang, S. , M. Li , D. Jin , and Y. Xie . 2023. “Inhibitory Effect of Blueberry Anthocyanins on Human Gastric Adenocarcinoma Cells and Transplanted Tumor in Nude Mice by Regulating the Endogenous Apoptosis Pathway.” Indian Journal of Pharmaceutical Sciences 85, no. 5: 1444–1451.

[fsn370232-bib-0054] Huang, Y. , M. An , A. Fang , O. J. Olatunji , and F. N. Eze . 2022. “Antiproliferative Activities of the Lipophilic Fraction of *Eucalyptus camaldulensis* Against MCF‐7 Breast Cancer Cells, UPLC‐ESI‐QTOF‐MS Metabolite Profile, and Antioxidative Functions.” ACS Omega 7, no. 31: 27369–27381. 35967023 10.1021/acsomega.2c02389 PMC9366772

[fsn370232-bib-0055] Ichiyanagi, T. , Y. Shida , M. M. Rahman , Y. Hatano , and T. Konishi . 2006. “Bioavailability and Tissue Distribution of Anthocyanins in Bilberry ( *Vaccinium myrtillus* L.) Extract in Rats.” Journal of Agricultural and Food Chemistry 54, no. 18: 6578–6587. 16939312 10.1021/jf0602370

[fsn370232-bib-0056] Jebir, R. M. , and Y. F. Mustafa . 2022. “Watermelon Allsweet: A Promising Natural Source of Bioactive Products.”

[fsn370232-bib-0057] Jing, N. , J. Song , Z. Liu , L. Wang , and G. Jiang . 2020. “Glycosylation of Anthocyanins Enhances the Apoptosis of Colon Cancer Cells by Handicapping Energy Metabolism.” BMC Complementary Medicine and Therapies 20, no. 1: 1–13. 10.1186/s12906-020-03096-y. 33059637 PMC7566133

[fsn370232-bib-0058] Jongsomchai, K. , V. Leardkamolkarn , and S. Mahatheeranont . 2020. “A Rice Bran Phytochemical, Cyanidin 3‐Glucoside, Inhibits the Progression of PC3 Prostate Cancer Cell.” Anatomy & Cell Biology 53, no. 4: 481–492. 32839357 10.5115/acb.20.085 PMC7769112

[fsn370232-bib-0059] Kanokpanont, S. , R. Yamdech , and P. Aramwit . 2018. “Stability Enhancement of Mulberry‐Extracted Anthocyanin Using Alginate/Chitosan Microencapsulation for Food Supplement Application.” Artificial Cells, Nanomedicine, and Biotechnology 46, no. 4: 773–782. 10.1080/21691401.2017.1339050 28599580

[fsn370232-bib-0060] Karakaş, N. , M. E. Okur , T. Sağır , D. Uludağ , D. Ç. Polat , and A. E. Karadağ . 2022. “Antioxidant Activity and Anti‐Cancer Effects of Bilberry (*Vaccinium myrtillus* L.) Fruit Extract on Gastric Cancer, AGS Cell Line.” Journal of Faculty of Pharmacy of Ankara University 46, no. 3: 781–792.

[fsn370232-bib-0061] Kausar, H. , J. Jeyabalan , F. Aqil , et al. 2012. “Berry Anthocyanidins Synergistically Suppress Growth and Invasive Potential of Human Non‐Small‐Cell Lung Cancer Cells.” Cancer Letters 325, no. 1: 54–62. 22659736 10.1016/j.canlet.2012.05.029

[fsn370232-bib-0062] Kido, L. A. , I. M. U. Rossetto , A. M. Baseggio , et al. 2022. “Brazilian Berry Extract Differentially Induces Inflammatory and Immune Responses in Androgen Dependent and Independent Prostate Cancer Cells.” Journal of Cancer Prevention 27, no. 3: 182–191. 10.15430/JCP.2022.27.3.182. 36258714 PMC9537582

[fsn370232-bib-0063] Kim, H. J. , B. H. Kim , B. R. Jin , S. J. Park , and H. J. An . 2022. “Purple Corn Extract Improves Benign Prostatic Hyperplasia by Regulating Prostate Cell Proliferation and Apoptosis.” Journal of Agricultural and Food Chemistry 70, no. 18: 5561–5569. 35466676 10.1021/acs.jafc.1c07955

[fsn370232-bib-0064] Kim, M. H. , S. Park , G. Song , W. Lim , and Y. S. Han . 2022. “Antigrowth Effects of Kaempferia Parviflora Extract Enriched in Anthocyanidins on Human Ovarian Cancer Cells Through Ca^2+^‐ROS Overload and Mitochondrial Dysfunction.” Molecular & Cellular Toxicology 18, no. 3: 383–391.

[fsn370232-bib-0065] Kim, M. J. , A. Paramanantham , W. S. Lee , et al. 2020. “Anthocyanins Derived From Vitis Coignetiae Pulliat Contributes Anti‐Cancer Effects by Suppressing NF‐κB Pathways in Hep3B Human Hepatocellular Carcinoma Cells and *In Vivo* .” Molecules 25, no. 22: 5445. 10.3390/molecules25225445. 33233701 PMC7699833

[fsn370232-bib-0066] Kim, N. H. , J. Jegal , Y. N. Kim , et al. 2020. “The Effects of *Aronia melanocarpa* Extract on Testosterone‐Induced Benign Prostatic Hyperplasia in Rats, and Quantitative Analysis of Major Constituents Depending on Extract Conditions.” Nutrients 12, no. 6: 1575. 32481550 10.3390/nu12061575 PMC7352698

[fsn370232-bib-0067] Kirakosyan, A. , E. M. Seymour , J. Wolforth , R. McNish , P. B. Kaufman , and S. F. Bolling . 2015. “Tissue Bioavailability of Anthocyanins From Whole Tart Cherry in Healthy Rats.” Food Chemistry 171: 26–31. 25308638 10.1016/j.foodchem.2014.08.114

[fsn370232-bib-0068] Kohut, L. , S. Baldovska , O. Paulen , M. Mihal , and A. Kolesarova . 2022. “The Assessment of Modulatory Effects of Blackcurrant (*Ribes nigrum* L.) and Chokeberry (*Aronia melanocarpa* L.) on Ovarian Cell Functions *In Vitro* .” Journal of Microbiology, Biotechnology and Food Sciences 12, no. 3: e9671. 10.55251/jmbfs.9671.

[fsn370232-bib-0069] Lage, N. N. , M. A. A. Layosa , S. Arbizu , et al. 2020. “Dark Sweet Cherry (*Prunus avium*) Phenolics Enriched in Anthocyanins Exhibit Enhanced Activity Against the Most Aggressive Breast Cancer Subtypes Without Toxicity to Normal Breast Cells.” Journal of Functional Foods 64: 103710. 10.1016/j.jff.2019.103710.

[fsn370232-bib-0070] Lakkis, N. A. , R. M. Abdallah , U. M. Musharrafieh , H. G. Issa , and M. H. Osman . 2024. “Epidemiology of Breast, Corpus Uteri, and Ovarian Cancers in Lebanon With Emphasis on Breast Cancer Incidence Trends and Risk Factors Compared to Regional and Global Rates.” Cancer Control 31: 10732748241236266. 38419342 10.1177/10732748241236266 PMC10903209

[fsn370232-bib-0071] Lang, Y. , J. Tian , X. Meng , et al. 2021. “Effects of α‐Casein on the Absorption of Blueberry Anthocyanins and Metabolites in Rat Plasma Based on Pharmacokinetic Analysis.” Journal of Agricultural and Food Chemistry 69, no. 22: 6200–6213. 10.1021/acs.jafc.1c00082. 34044544

[fsn370232-bib-0072] Layosa, M. A. A. , N. N. Lage , B. P. Chew , et al. 2021. “Dark Sweet Cherry ( *Prunus avium* ) Phenolics Enriched in Anthocyanins Induced Apoptosis in Mda‐Mb‐453 Breast Cancer Cells Through Mapk‐Dependent Signaling and Reduced Invasion via Akt and Plcγ‐1 Downregulation.” Nutrition and Cancer 73, no. 10: 1985–1997. 32924599 10.1080/01635581.2020.1817514

[fsn370232-bib-0073] Li, S. , X. Li , H. Xu , et al. 2024. “Alternating Modified CAPOX/CAPIRI Plus Bevacizumab in Untreated Unresectable Metastatic Colorectal Cancer: A Phase 2 Trial.” Signal Transduction and Targeted Therapy 9, no. 1: 1–7. 39658608 10.1038/s41392-024-02048-z PMC11631963

[fsn370232-bib-0074] Li, X. , J. Mu , and X. Meng . 2021. Anti‐Tumour Effect of Cyanidin‐3‐o‐glucoside Combined With Cisplatin in the Mice Xenograft Models of Cervical Cancer.

[fsn370232-bib-0075] Li, X. , J. Zhao , T. Yan , et al. 2021. “Cyanidin‐3‐O‐Glucoside and Cisplatin Inhibit Proliferation and Downregulate the PI3K/AKT/mTOR Pathway in Cervical Cancer Cells.” Journal of Food Science 86, no. 6: 2700–2712. 10.1111/1750-3841.15740. 33908630

[fsn370232-bib-0076] Li, Z. L. , J. Mi , L. Lu , et al. 2021. “The Main Anthocyanin Monomer of Lycium Ruthenicum Murray Induces Apoptosis Through the ROS/PTEN/PI3K/Akt/Caspase 3 Signaling Pathway in Prostate Cancer DU‐145 Cells.” Food & Function 12, no. 4: 1818–1828. 10.1039/d0fo02382e. 33527955

[fsn370232-bib-0077] Liu, L. , T. Zhao , H. Yang , Z. Hu , and C. Xie . 2024. “Cyanidin‐3‐O‐Glucoside Combined With PGC1α Inhibitor Promotes Cancer Cell Death by Inducing Excessive Levels of ROS in Cervical Squamous Cell Carcinoma.” Journal of Food Biochemistry 2024, no. 1: 4953859.

[fsn370232-bib-0078] Liu, X. , D. Zhang , Y. Hao , et al. 2018. “Cyanidin curtails renal cell carcinoma tumorigenesis.” Cellular Physiology and Biochemistry 46, no. 6: 2517–2531. 10.1159/000489658. 29742507

[fsn370232-bib-0079] Liu, Y. Y. , T. T. Gong , Y. Z. Li , et al. 2023. “Association of Pre‐Diagnosis Specific Color Groups of Fruit and Vegetable Intake With Ovarian Cancer Survival: Results From the Ovarian Cancer Follow‐Up Study (OOPS).” Food & Function 14, no. 18: 8442–8452. 10.1039/D3FO01443F. 37622277

[fsn370232-bib-0080] Lu, J. N. , R. Panchanathan , W. S. Lee , et al. 2017. “Anthocyanins From the Fruit of Vitis Coignetiae Pulliat Inhibit Tnf‐Augmented Cancer Proliferation, Migration, and Invasion in a549 Cells.” Asian Pacific Journal of Cancer Prevention: APJCP 18, no. 11: 2919–2923. 10.22034/APJCP.2017.18.11.2919. 29172259 PMC5773771

[fsn370232-bib-0081] Luo, H. , M. Gao , H. Lu , Q. Chen , and X. Lian . 2024. “Anthocyanins Prevent the Development and Progression of Urethane‐Induced Lung Cancer by Regulating Energy Metabolism in Mice.” Food & Nutrition Research 68. 10.29219/fnr.v68.10434. PMC11075467 38716355

[fsn370232-bib-0082] Ma, Y. , Y. Feng , T. Diao , W. Zeng , and Y. Zuo . 2020. “Experimental and Theoretical Study on Antioxidant Activity of the Four Anthocyanins.” Journal of Molecular Structure 1204: 127509.

[fsn370232-bib-0083] Macis, D. , I. M. Briata , O. D'Ecclesiis , et al. 2023. “Inflammatory and Metabolic Biomarker Assessment in a Randomized Presurgical Trial of Curcumin and Anthocyanin Supplements in Patients With Colorectal Adenomas.” Nutrients 15, no. 18: 3894. 10.3390/nu15183894. 37764678 PMC10537228

[fsn370232-bib-0084] Madanakumar, A. J. , B. Lawarence , G. S. Manoj , and M. Kumaraswamy . 2018. “Purified Anthocyanin From *In Vitro* Culture of *Bridelia retusa* (L.) Spreng. Capable of Inhibiting the Growth of Human Oral Squamous Cell Carcinoma Cells.” Pharmacognosy Journal 10, no. 3: 559–566.

[fsn370232-bib-0085] Matboli, M. , A. H. Hasanin , R. Hussein , et al. 2021. “Cyanidin 3‐Glucoside Modulated Cell Cycle Progression in Liver Precancerous Lesion, *In Vivo* Study.” World Journal of Gastroenterology 27, no. 14: 1435–1450. 33911466 10.3748/wjg.v27.i14.1435 PMC8047539

[fsn370232-bib-0086] Mauramo, M. , T. Onali , W. Wahbi , et al. 2021. “Bilberry (*Vaccinium myrtillus* L.) Powder Has Anticarcinogenic Effects on Oral Carcinoma *In Vitro* and *In Vivo* .” Antioxidants (Basel) 10, no. 8: 1319. 10.3390/antiox10081319. 34439567 PMC8389301

[fsn370232-bib-0087] McGlynn, K. A. , J. L. Petrick , and J. D. Groopman . 2024. “Liver Cancer: Progress and Priorities.” Cancer Epidemiology, Biomarkers & Prevention 33, no. 10: 1261–1272. 10.1158/1055-9965.EPI-24-0686 39354815

[fsn370232-bib-0088] Meena, K. , S. Kumari , S. Mishra , M. Saini , and J. S. Chauhan . 2024. “The Role of Genetics and Hormones in Women's Health.” In Women's Health: A Comprehensive Guide to Common Health Issues in Women, 74–100. Bentham Science Publishers.

[fsn370232-bib-0090] Mora, N. , M. Rosa , M. Touaibia , and L. J. Martin . 2024. “Effects of Red Sorghum‐Derived Deoxyanthocyanidins and Their O‐β‐D‐Glucosides on E‐Cadherin Promoter Activity in PC‐3 Prostate Cancer Cells.” Molecules 29, no. 8: 1891. 38675711 10.3390/molecules29081891 PMC11054106

[fsn370232-bib-0091] Mottaghipisheh, J. , A. H. Doustimotlagh , C. Irajie , N. Tanideh , A. Barzegar , and A. Iraji . 2022. “The Promising Therapeutic and Preventive Properties of Anthocyanidins/Anthocyanins on Prostate Cancer.” Cells 11, no. 7: 1070. 35406634 10.3390/cells11071070 PMC8997497

[fsn370232-bib-0092] Mueller, D. , K. Jung , M. Winter , et al. 2018. “Encapsulation of Anthocyanins From Bilberries–Effects on Bioavailability and Intestinal Accessibility in Humans.” Food Chemistry 248: 217–224. 10.1016/j.foodchem.2017.12.058. 29329847

[fsn370232-bib-0093] Mueller, D. , K. Jung , M. Winter , D. Rogoll , R. Melcher , and E. Richling . 2017. “Human Intervention Study to Investigate the Intestinal Accessibility and Bioavailability of Anthocyanins From Bilberries.” Food Chemistry 231: 275–286. 28450007 10.1016/j.foodchem.2017.03.130

[fsn370232-bib-0094] Munteanu, C. , and B. Schwartz . 2024. “Interactions Between Dietary Antioxidants, Dietary Fiber and the Gut Microbiome: Their Putative Role in Inflammation and Cancer.” International Journal of Molecular Sciences 25, no. 15: 8250. 39125822 10.3390/ijms25158250 PMC11311432

[fsn370232-bib-0095] Muscolo, A. , O. Mariateresa , T. Giulio , and R. Mariateresa . 2024. “Oxidative Stress: The Role of Antioxidant Phytochemicals in the Prevention and Treatment of Diseases.” International Journal of Molecular Sciences 25, no. 6: 3264. 38542238 10.3390/ijms25063264 PMC10970659

[fsn370232-bib-0096] Mustafa, Y. F. 2024. “Harmful Free Radicals in Aging: A Narrative Review of Their Detrimental Effects on Health.” Indian Journal of Clinical Biochemistry 39, no. 2: 154–167. 38577147 10.1007/s12291-023-01147-y PMC10987461

[fsn370232-bib-0097] Mustafa, Y. F. , A. F. Faisal , M. M. Alshaher , and D. A. Hassan . 2025. “Food‐Derived Micronutrients as Alleviators of Age‐Related Dysfunction: A Dive Into Their Effects and Cellular Mechanisms.” Indian Journal of Clinical Biochemistry: 1–17. 10.1007/s12291-024-01297-7.39835227

[fsn370232-bib-0098] Nedungadi, D. , N. Ryan , K. Anderson , et al. 2022. “Modulation of the Oral Glucocorticoid System During Black Raspberry Mediated Oral Cancer Chemoprevention.” Carcinogenesis 43, no. 1: 28–39. 10.1093/carcin/bgab118. 34888650 PMC8832455

[fsn370232-bib-0099] Oda, B. K. , E. Lulekal , B. Warkineh , Z. Asfaw , and A. Debella . 2024. “Ethnoveterinary Medicinal Plants and Their Utilization by Indigenous and Local Communities of Dugda District, Central Rift Valley, Ethiopia.” Journal of Ethnobiology and Ethnomedicine 20, no. 1: 32. 10.1186/s13002-024-00665-0. 38461267 PMC10924356

[fsn370232-bib-0100] Oh, C. K. , and Y. S. Cho . 2024. “Pathogenesis and Biomarkers of Colorectal Cancer by Epigenetic Alteration.” Intestinal Research 22, no. 2: 131–151. 38295766 10.5217/ir.2023.00115 PMC11079515

[fsn370232-bib-0101] Olejnik, A. , M. Kaczmarek , M. Olkowicz , K. Kowalska , W. Juzwa , and R. Dembczyński . 2018. “ROS‐Modulating Anticancer Effects of Gastrointestinally Digested *Ribes nigrum* L. Fruit Extract in Human Colon Cancer Cells.” Journal of Functional Foods 42: 224–236.

[fsn370232-bib-0102] Pal, H. C. , S. Sharma , L. R. Strickland , et al. 2013. “Delphinidin Reduces Cell Proliferation and Induces Apoptosis of Non‐Small‐Cell Lung Cancer Cells by Targeting EGFR/VEGFR2 Signaling Pathways.” PLoS One 8, no. 10: e77270. 24124611 10.1371/journal.pone.0077270 PMC3790876

[fsn370232-bib-0103] Pandey, J. , and W. Syed . 2024. “Renal Cancer.” In StatPearls. StatPearls Publishing. 32644401

[fsn370232-bib-0104] Paramanantham, A. , M. J. Kim , E. J. Jung , et al. 2020. “Anthocyanins Isolated From Vitis Coignetiae Pulliat Enhances Cisplatin Sensitivity in MCF‐7 Human Breast Cancer Cells Through Inhibition of Akt and NF‐κB Activation.” Molecules 25, no. 16: 3623. 10.3390/molecules25163623. 32784919 PMC7466154

[fsn370232-bib-0105] Park, C. , W. S. Lee , S. I. Go , et al. 2021. “Apoptotic Effects of Anthocyanins From Vitis Coignetiae Pulliat Are Enhanced by Augmented Enhancer of the Rudimentary Homolog (ERH) in Human Gastric Carcinoma MKN28 Cells.” International Journal of Molecular Sciences 22, no. 6: 3030. 10.3390/ijms22063030. 33809701 PMC8002340

[fsn370232-bib-0106] Park, S. H. , M. Kim , S. Lee , W. Jung , and B. Kim . 2021. “Therapeutic Potential of Natural Products in Treatment of Cervical Cancer: A Review.” Nutrients 13, no. 1: 154. 33466408 10.3390/nu13010154 PMC7824868

[fsn370232-bib-0107] Pieńkowska, N. , G. Bartosz , P. Furdak , and I. Sadowska‐Bartosz . 2021. “Delphinidin Increases the Sensitivity of Ovarian Cancer Cell Lines to 3‐Bromopyruvate.” International Journal of Molecular Sciences 22, no. 2: 709. 33445795 10.3390/ijms22020709 PMC7828231

[fsn370232-bib-0108] Prakash, S. , K. Radha , M. Kumar , et al. 2021. “Plant‐Based Antioxidant Extracts and Compounds in the Management of Oral Cancer.” Antioxidants (Basel) 10, no. 9: 1358. 34572990 10.3390/antiox10091358 PMC8466097

[fsn370232-bib-0109] Ribera‐Fonseca, A. , D. Jiménez , P. Leal , et al. 2020. “The Anti‐Proliferative and Anti‐Invasive Effect of Leaf Extracts of Blueberry Plants Treated With Methyl Jasmonate on Human Gastric Cancer *In Vitro* Is Related to Their Antioxidant Properties.” Antioxidants 9, no. 1: 45. 10.3390/antiox9010045. 31948009 PMC7023271

[fsn370232-bib-0110] Romualdo, G. R. , I. P. de Souza , L. V. de Souza , et al. 2021. “Beneficial effects of anthocyanin‐rich peels of Myrtaceae fruits on chemically‐induced liver fibrosis and carcinogenesis in mice.” Food Research International 139: 109964. 33509514 10.1016/j.foodres.2020.109964

[fsn370232-bib-0111] Salah, M. , M. Mansour , D. Zogona , and X. Xu . 2020. “Nanoencapsulation of Anthocyanins‐Loaded β‐Lactoglobulin Nanoparticles: Characterization, Stability, and Bioavailability *In Vitro* .” Food Research International 137: 109635. 33233214 10.1016/j.foodres.2020.109635

[fsn370232-bib-0112] Samanci, A. E. T. , N. B. Muluk , T. Samanci , and C. Cingi . 2024. “Propolis: Prevention and Healing Effects in Otorhinolaryngology.”

[fsn370232-bib-0113] Santos, T. S. , M. O. Bahia , A. C. Guimarães , et al. 2024. “ *In Vitro* Assessment of the Genotoxic and Cytotoxic Effects of Clarified açai (*Euterpe oleracea* MART) Extract in a Gastric Cancer Cell Line (AGP01 Cells).” Toxicology In Vitro 99: 105873. 10.1016/j.tiv.2024.105873. 38851601

[fsn370232-bib-0114] Schmutz, C. , F. Will , E. Varga , et al. 2023. “ *In Vitro* Inhibitory Potential of Different Anthocyanin‐Rich Berry Extracts in Murine CT26 Colon Cancer Cells.” Molecules 28, no. 23: 7684. 38067418 10.3390/molecules28237684 PMC10707341

[fsn370232-bib-0115] Silveira Rabelo, A. C. , S. U. Mertens‐Talcott , B. P. Chew , and G. Noratto . 2022. “Dark Sweet Cherry ( *Prunus avium* ) Anthocyanins Suppressed ERK1/2‐Akt/mTOR Cell Signaling and Oxidative Stress: Implications for TNBC Growth and Invasion.” Molecules 27, no. 21: 7245. 36364072 10.3390/molecules27217245 PMC9657292

[fsn370232-bib-0116] Simas Frauches, N. , J. Montenegro , T. Amaral , et al. 2021. “Antiproliferative Activity on Human Colon Adenocarcinoma Cells and *In Vitro* Antioxidant Effect of Anthocyanin‐Rich Extracts From Peels of Species of the Myrtaceae Family.” Molecules 26, no. 3: 564. 33498977 10.3390/molecules26030564 PMC7865521

[fsn370232-bib-0117] Sirilun, S. , C. Chaiyasut , T. Pattananandecha , et al. 2022. “Enhancement of the Colorectal Chemopreventive and Immunization Potential of Northern Thai Purple Rice Anthocyanin Using the Biotransformation by β‐Glucosidase‐Producing Lactobacillus.” Antioxidants 11, no. 2: 305. 35204188 10.3390/antiox11020305 PMC8868395

[fsn370232-bib-0118] Sun, W. , N. D. Zhang , T. Zhang , et al. 2023. “Cyanidin‐3‐O‐Glucoside Induces the Apoptosis of Human Gastric Cancer MKN‐45 Cells Through ROS‐Mediated Signaling Pathways.” Molecules 28, no. 2: 652. 10.3390/molecules28020652. 36677726 PMC9860697

[fsn370232-bib-0119] Sundarrajan, P. , and L. Bhagtaney . 2024. “Traditional Medicinal Plants as Bioresources in Health Security.” In Ethnic Knowledge and Perspectives of Medicinal Plants, 53–75. Apple Academic Press.

[fsn370232-bib-0120] Tan, J. , Q. Li , H. Xue , and J. Tang . 2020. “Ultrasound‐Assisted Enzymatic Extraction of Anthocyanins From Grape Skins: Optimization, Identification, and Antitumor Activity.” Journal of Food Science 85, no. 11: 3731–3744. 33078395 10.1111/1750-3841.15497

[fsn370232-bib-0121] Teniente, S. L. , A. C. Flores‐Gallegos , S. C. Esparza‐González , L. G. Campos‐Múzquiz , S. D. Nery‐Flores , and R. Rodríguez‐Herrera . 2023. “Anticancer Effect of Pomegranate Peel Polyphenols Against Cervical Cancer.” Antioxidants 12, no. 1: 127. 36670990 10.3390/antiox12010127 PMC9854619

[fsn370232-bib-0122] Tikhonova, M. A. , O. Y. Shoeva , M. V. Tenditnik , et al. 2024. “Antitumor Effects of an Anthocyanin‐Rich Grain Diet in a Mouse Model of Lewis Lung Carcinoma.” International Journal of Molecular Sciences 25, no. 11: 5727. 10.3390/ijms25115727. 38891915 PMC11171629

[fsn370232-bib-0123] Tsai, M. C. , C. C. Chen , T. H. Tseng , et al. 2023. “Hibiscus Anthocyanins Extracts Induce Apoptosis by Activating AMP‐Activated Protein Kinase in Human Colorectal Cancer Cells.” Nutrients 15, no. 18: 3972. 10.3390/nu15183972. 37764756 PMC10535221

[fsn370232-bib-0124] Tsuyuki, S. , S. Fukui , A. Watanabe , S. Akune , M. Tanabe , and K. Yoshida . 2012. “Delphinidin Induces Autolysosome as Well as Autophagosome Formation and Delphinidin‐Induced Autophagy Exerts a Cell Protective Role.” Journal of Biochemical and Molecular Toxicology 26, no. 11: 445–453. 23129091 10.1002/jbt.21443

[fsn370232-bib-0125] Ullah, A. , M. Arif , M. Waqas , et al. 2024. “Exploring the Role of Chemicals and Environmental Factors for Cancer Proliferation.” Open Access Library Journal 11, no. 12: 1–19.

[fsn370232-bib-0126] Vázquez‐Lorente, H. , L. Herrera‐Quintana , L. Jiménez‐Sánchez , B. Fernández‐Perea , and J. Plaza‐Diaz . 2024. “Antioxidant Functions of Vitamin D and CYP11A1‐Derived Vitamin D, Tachysterol, and Lumisterol Metabolites: Mechanisms, Clinical Implications, and Future Directions.” Antioxidants 13, no. 8: 996. 39199241 10.3390/antiox13080996 PMC11351441

[fsn370232-bib-0127] Vishnu, V. R. , R. S. Renjith , A. Mukherjee , S. R. Anil , J. Sreekumar , and A. N. Jyothi . 2019. “Comparative Study on the Chemical Structure and *In Vitro* Antiproliferative Activity of Anthocyanins in Purple Root Tubers and Leaves of Sweet Potato ( *Ipomoea batatas* ).” Journal of Agricultural and Food Chemistry 67, no. 9: 2467–2475. 30741542 10.1021/acs.jafc.8b05473

[fsn370232-bib-0128] Wang, H. , Q. Jia , J. Jiang , and L. Huang . 2023. “Ultrasound Assisted Aqueous Two‐Phase Extraction of Anthocyanins From Blueberry and Its Anti‐Tumor Activity.” Food Science and Technology Research 29, no. 4: 319–330.

[fsn370232-bib-0129] Wang, W. , J. Jung , and Y. Zhao . 2017. “Chitosan‐Cellulose Nanocrystal Microencapsulation to Improve Encapsulation Efficiency and Stability of Entrapped Fruit Anthocyanins.” Carbohydrate Polymers 157: 1246–1253. 27987829 10.1016/j.carbpol.2016.11.005

[fsn370232-bib-0130] Wang, Y. , X. Tian , T. Cheng , R. Liu , and F. Han . 2024. “Anthocyanins and Proanthocyanidins Synergistically Inhibit the Growth of Gastric Cancer Cells *In Vitro*: Exploring the Potential Physiological Activity of Grape and Red Wine.” Natural Product Research: 1–10. 10.1080/14786419.2024.2373957. 38956986

[fsn370232-bib-0131] Wang, Y. , Y. Ye , L. Wang , W. Yin , and J. Liang . 2021. “Antioxidant Activity and Subcritical Water Extraction of Anthocyanin From Raspberry Process Optimization by Response Surface Methodology.” Food Bioscience 44: 101394.

[fsn370232-bib-0132] Wei, J. , H. Wu , H. Zhang , et al. 2018. “Anthocyanins Inhibit High Glucose‐Induced Renal Tubular Cell Apoptosis Caused by Oxidative Stress in Db/Db Mice.” International Journal of Molecular Medicine 41, no. 3: 1608–1618. 10.3892/ijmm.2018.3378. 29328429 PMC5819916

[fsn370232-bib-0133] Wu, T. , E. Lucas , F. Zhao , P. Basu , and Y. Qiao . 2024. “Artificial Intelligence Strengthenes Cervical Cancer Screening–Present and Future.” Cancer Biology & Medicine 21, no. 10: 864. 39297572 10.20892/j.issn.2095-3941.2024.0198 PMC11523278

[fsn370232-bib-0134] Xiao, T. , Z. Luo , Z. Guo , et al. 2021. “Multiple Roles of Black Raspberry Anthocyanins Protecting Against Alcoholic Liver Disease.” Molecules 26, no. 8: 2313. 10.3390/molecules26082313. 33923467 PMC8073606

[fsn370232-bib-0135] Xie, L. , S. G. Lee , T. M. Vance , et al. 2016. “Bioavailability of Anthocyanins and Colonic Polyphenol Metabolites Following Consumption of Aronia Berry Extract.” Food Chemistry 211: 860–868. 10.1016/j.foodchem.2016.05.122. 27283706

[fsn370232-bib-0136] Xu, X. , Y. Zhu , S. Li , and D. Xia . 2023. “Dietary Intake of Anthocyanidins and Renal Cancer Risk: A Prospective Study.” Cancers 15, no. 5: 1406. 36900199 10.3390/cancers15051406 PMC10001018

[fsn370232-bib-0137] Yang, D. , Y. Ran , X. Li , et al. 2022. “Cyanidin‐3‐O‐Glucoside Ameliorates Cadmium Induced Uterine Epithelium Proliferation in Mice.” Journal of Hazardous Materials 425: 127571. 10.1016/j.jhazmat.2021.127571. 34986559

[fsn370232-bib-0138] Yang, S. T. , P. H. Wang , H. H. Liu , C. W. Chang , W. H. Chang , and W. L. Lee . 2024. “Cervical Cancer: Part II the Landscape of Treatment for Persistent, Recurrent and Metastatic Diseases (I).” Taiwanese Journal of Obstetrics & Gynecology 63, no. 5: 637–650. 39266144 10.1016/j.tjog.2024.08.001

[fsn370232-bib-0139] Yeewa, R. , A. Naiki‐Ito , T. Naiki , et al. 2020. “Hexane Insoluble Fraction From Purple Rice Extract Retards Carcinogenesis and Castration‐Resistant Cancer Growth of Prostate Through Suppression of Androgen Receptor Mediated Cell Proliferation and Metabolism.” Nutrients 12, no. 2: 558. 32093357 10.3390/nu12020558 PMC7071398

[fsn370232-bib-0140] Younes, A. H. , and Y. F. Mustafa . 2024. “Novel Coumarins From Green Sweet Bell Pepper Seeds: Their Isolation, Characterization, Oxidative Stress‐Mitigating, Anticancer, Anti‐Inflammatory, and Antidiabetic Properties.” Journal of Molecular Structure 1312: 138629.

[fsn370232-bib-0141] Yu, W. , J. Gao , R. Hao , et al. 2021. “ *Aronia melanocarpa* Elliot Anthocyanins Inhibit Colon Cancer by Regulating Glutamine Metabolism.” Food Bioscience 40: 100910.

[fsn370232-bib-0142] Yuan, Y. , Q. Fan , X. Xu , O. Wang , L. Zhao , and L. Zhao . 2023. “Nanocarriers Based on Polysaccharides for Improving the Stability and Bioavailability of Anthocyanins: A Review.” Carbohydrate Polymer Technologies and Applications 6: 100346.

[fsn370232-bib-0143] Yücetepe, M. , Z. T. Özaslan , M. Ş. Karakuş , et al. 2024. “Unveiling the Multifaceted World of Anthocyanins: Biosynthesis Pathway, Natural Sources, Extraction Methods, Copigmentation, Encapsulation Techniques, and Future Food Applications.” Food Research International 187: 114437. 10.1016/j.foodres.2024.114437. 38763684

[fsn370232-bib-0144] Yue, E. , G. Tuguzbaeva , X. Chen , et al. 2019. “Anthocyanin Is Involved in the Activation of Pyroptosis in Oral Squamous Cell Carcinoma.” Phytomedicine 56: 286–294. 10.1016/j.phymed.2018.09.223. 30668350

[fsn370232-bib-0145] Zang, Z. , S. Chou , L. Geng , et al. 2021. “Interactions of Blueberry Anthocyanins With Whey Protein Isolate and Bovine Serum Protein: Color Stability, Antioxidant Activity, *In Vitro* Simulation, and Protein Functionality.” Lwt 152: 112269. 10.1016/j.lwt.2021.112269.

[fsn370232-bib-0146] Zannou, O. , and I. Koca . 2022. “Greener Extraction of Anthocyanins and Antioxidant Activity From Blackberry (*Rubus* spp.) Using Natural Deep Eutectic Solvents.” Lwt 158: 113184.

[fsn370232-bib-0147] Zannou, O. , H. Pashazadeh , M. Ghellam , S. A. Ibrahim , and I. Koca . 2021. “Extraction of Anthocyanins From Borage (*Echium amoenum*) Flowers Using Choline Chloride and a Glycerol‐Based, Deep Eutectic Solvent: Optimization, Antioxidant Activity, and *In Vitro* Bioavailability.” Molecules 27, no. 1: 134. 10.3390/molecules27010134. 35011365 PMC8746641

[fsn370232-bib-0148] Zhang, G. , Y. Jiang , X. Liu , Y. Deng , B. Wei , and L. Shi . 2021. “Lingonberry Anthocyanins Inhibit Hepatic Stellate Cell Activation and Liver Fibrosis via tgfβ/Smad/ERK Signaling Pathway.” Journal of Agricultural and Food Chemistry 69, no. 45: 13546–13556. 34735147 10.1021/acs.jafc.1c05384

[fsn370232-bib-0149] Zhang, Q. , W. Yang , J. Liu , et al. 2021. “Postharvest UV‐C Irradiation Increased the Flavonoids and Anthocyanins Accumulation, Phenylpropanoid Pathway Gene Expression, and Antioxidant Activity in Sweet Cherries (*Prunus avium* L.).” Postharvest Biology and Technology 175: 111490. 10.1016/j.postharvbio.2021.111490.

[fsn370232-bib-0150] Zhang, Y. , M. Zhu , H. Wan , L. Chen , and F. Luo . 2022. “Association Between Dietary Anthocyanidins and Risk of Lung Cancer.” Nutrients 14, no. 13: 2643. 35807824 10.3390/nu14132643 PMC9268346

[fsn370232-bib-0151] Zhao, F. , J. Wang , W. Wang , L. Lyu , W. Wu , and W. Li . 2023. “The Extraction and High Antiproliferative Effect of Anthocyanin From Gardenblue Blueberry.” Molecules 28, no. 6: 2850. 36985822 10.3390/molecules28062850 PMC10054926

